# Paralimibaculum aggregatum gen. nov. sp. nov. and Biformimicrobium ophioploci gen. nov. sp. nov., two novel heterotrophs from brittle star Ophioplocus japonicus

**DOI:** 10.1099/ijsem.0.006530

**Published:** 2024-09-26

**Authors:** Keisuke Kawano, Tatsuya Awano, Arata Yoshinaga, Junji Sugiyama, Shigeki Sawayama, Satoshi Nakagawa

**Affiliations:** 1 Laboratory of Marine Environmental Microbiology, Division of Applied Biosciences, Graduate School of Agriculture, Kyoto University, Oiwake-cho, Kitashirakawa, Sakyo-ku, Kyoto 606-8502, Japan; 2 Laboratory of Tree Cell Biology, Division of Forest and Biomaterials Science, Graduate School of Agriculture, Kyoto University, Oiwake-cho, Kitashirakawa, Sakyo-ku, Kyoto 606-8502, Japan; 3 Institute for Extra-Cutting-Edge Science and Technology Avant-Garde Research (X-Star), Japan Agency for Marine-Earth Science & Technology (JAMSTEC), 2-15 Natsushima-Cho, Yokosuka 237-0061, Japan; 4 Section for Exploration of Life in Extreme Environments, Exploratory Research Center on Life and Living Systems (ExCELLS), National Institute of Natural Sciences, 5-1 Higashiyama, Myodaiji-Cho, Okazaki 444-8787, Japan

**Keywords:** brittle star, *Microbulbiferaceae*, *Paracoccaceae*, symbiosis, tidal flat

## Abstract

Two novel Gram-stain-negative, strictly aerobic, halophilic and non-motile bacterial strains, designated NKW23^T^ and NKW57^T^, were isolated from a brittle star *Ophioplocus japonicus* collected from a tidal pool in Wakayama, Japan. The results of phylogenetic analysis based on 16S rRNA gene sequences indicated that NKW23^T^ represented a member of the family *Paracoccaceae*, with *Limibaculum halophilum* CAU 1123^T^ as its closest relative (94.4% sequence identity). NKW57^T^ was identified as representing a member of the family *Microbulbiferaceae,* with up to 94.9% sequence identity with its closest relatives. Both strains displayed average nucleotide identity (ANI) and digital DNA–DNA hybridisation (dDDH) values below the species delimitation threshold against their closest relatives. Additionally, amino acid identity (AAI) values of both strains fell below the genus-defining threshold. Phylogenetic trees based on genome sequences indicated that NKW23^T^ formed a novel lineage, branching deeply prior to the divergence of the genera *Limibaculum* and *Thermohalobaculum,* with an evolutionary distance (ED) of 0.31–0.32, indicative of genus-level differentiation. NKW57^T^ similarly formed a distinct lineage separate from the species of the genus *Microbulbifer*. The major respiratory quinones of NKW23^T^ and NKW57^T^ were ubiquinone-10 (Q-10) and Q-8, respectively. The genomic DNA G+C contents of NKW23^T^ and NKW57^T^ were 71.4 and 58.8%, respectively. On the basis of the physiological and phylogenetic characteristics, it was proposed that these strains should be classified as novel species representing two novel genera: *Paralimibaculum aggregatum* gen. nov., sp. nov., with strain NKW23^T^ (=JCM 36220^T^=KCTC 8062^T^) as the type strain, and *Biformimicrobium ophioploci* gen. nov., sp. nov., with strain NKW57^T^ (=JCM 36221^T^=KCTC 8063^T^) as the type strain.

## Introduction

The family *Paracoccaceae,* which was reclassified from the family *Rhodobacteraceae*, currently comprises 69 genera (as of June 25, 2024, according to the LPSN) [1,2]. The major habitats of this family are widely distributed in various marine environments, such as marine sediments [3,4], seawater [5], coral [6] and ascidians [7]. The members of the family *Paracoccaceae* play an important role in marine ecosystems, including the degradation of xenobiotic aromatic compounds [8]. The genus *Limibaculum*, which is part of this family, included two species, *Limibaculum halophilum* and *Limibaculum sediminis*, but *L. sediminis* has been recently proposed to be reclassified as a member of the genus *Thermohalobaculum* [9]. Therefore, the genus *Limibaculum* currently includes only one unambiguously assigned species, *L. halophilum*, which was isolated from mud [10]. Members of the genus *Limibaculum* were predominantly detected on the surfaces of microplastics and seaweed [11,12].

The family *Microbulbiferaceae* is classified within the order *Cellvibrionales*, along with four other families: *Cellvibrionaceae*, *Porticoccaceae*, *Halieaceae* and *Spongiibacteraceae* [13]. The genus *Microbulbifer*, which represents the sole genus in the family *Microbulbiferaceae*, primarily includes members isolated from marine environments [14], marine organisms such as algae, sponges and sea urchins [15,17] and the rhizosphere of halophytes [18]. This genus encompasses 31 species with validly published names, with *Microbulbifer hydrolyticus* as the type species (as of June 25, 2024, according to the LPSN) [2,14]. Members of this genus play significant roles in marine ecosystems, particularly those known to degrade polysaccharides, such as chitin, alginic acid, galactan and agar [14,15, 19,21], and produce alkanoyl imidazoles with broad-spectrum antibacterial activities [22]. A notable characteristic of the members of this genus is the ability of some strains to undergo morphological transitions from rod-shaped to coccoid forms in response to environmental changes [15].

Echinoderms are known to host symbiotic bacteria in various body parts, including the gastrointestinal tract, surface, coelomic fluid and beneath the cuticle [23,28]. These bacteria exhibit significant differences from those found in the surrounding environment. Some of these symbiotic bacteria have garnered interest due to their ability to produce potentially beneficial secondary metabolites [27] and their contribution to the nutrition of their hosts [23,28]. However, the diversity of microbes associated with brittle stars has not been fully investigated. In this study, we isolated two novel strains, designated NKW23^T^ and NKW57^T^, from the brittle star *Ophioplocus japonicus* collected from a tidal pool in Wakayama, Japan. On the basis of the phenotypic, phylogenetic and chemotaxonomic characteristics, we propose that these strains represent two novel species belonging to newly proposed genera.

## Isolation of strains

The brittle star *Ophioplocus japonicus* specimens were collected from a tidal pool in Wakayama, Japan (34° 26’ N, 135° 06’ E) and transported to the laboratory with aeration. The surface of each specimen was washed with sterile artificial seawater (Instant Ocean, Aquarium Systems) to remove any debris. Subsequently, a portion of an arm was excised using sterilised dissecting scissors and placed in a 1.5 ml tube. These arm samples were homogenised in sterile artificial seawater using plastic pestles, followed by centrifugation at 860 ***g*** for 1 min at room temperature to remove the host skeletons and larger debris. The resulting supernatant served as the inoculum for marine broth 2216 (MB) agar plates. The plates were composed of MB medium solidified with 1.5% (w/v) agar. The MB medium consisted of the following components: 5.0 g l^−1^ peptone, 1.0 g l^−1^ yeast extract, 0.1 g l^−1^ ferric citrate, 19.45 g l^−1^ NaCl, 8.8 g l^−1^ MgCl_2_·6H_2_O, 3.24 g l^−1^ Na_2_SO_4_, 1.8 g l^−1^ CaCl_2_·2H_2_O, 0.55 g l^−1^ KCl, 0.16 g l^−1^ NaHCO_3_, 0.008 g l^−1^ KBr, 0.034 g l^−1^ SrCl_2_, 0.022 g l^−1^ H_3_BO_3_, 0.004 g l^−1^ Na_2_SiO_3_, 0.0024 g l^−1^ NaF, 0.0016 g l^−1^ NH_4_NO_3_ and 0.008 g l^−1^ Na_2_HPO_4_. The plates were incubated at 20 °C for 5 days. For purification, single colonies were successively streaked onto new agar plates three times. The pure cultures obtained were designated as strains NKW23^T^ and NKW57^T^. Reference strains *Limibaculum halophilum* CAU 1123^T^ and *Microbulbifer marinus* CGMCC 1.10657^T^ were obtained from the Korean Collection for Type Cultures (KCTC).

## 16S ribosomal RNA gene phylogeny

Genomic DNA was extracted using a DNA mini kit (Qiagen). PCR amplification of the 16S rRNA gene was performed using the Eubac 27F and 1492R primers [29], and it was sequenced by Macrogen Japan (Tokyo, Japan). Analysis of the 16S rRNA gene sequences of NKW23^T^ (1387 bases) and NKW57^T^ (1443 bases) was performed using the EzBioCloud platform (database version 2023.08.23) [30]. Multiple alignments of the 16S rRNA gene sequences were generated using MAFFT online version 7 [31]. Phylogenetic trees were reconstructed employing maximum-likelihood, neighbor-joining and maximum-parsimony methods, with the Tamura–Nei model and 1000 bootstrap replications, as facilitated by the mega 11 software package [32,33]. NKW23^T^ was found to be closely related to *Limibaculum halophilum* CAU 1123^T^, *Limibaculum sediminis* FT325^T^ and *Thermohalobaculum xanthum* M0105^T^, with sequence identities of 94.4, 93.1 and 94.2%, respectively. Thus, the name *T. xanthum* M0105^T^ published in June 2024 should not have been a valid name. The phylogenetic tree based on the 16S rRNA gene sequence revealed that NKW23^T^ and *Coraliihabitans acroporae* NNCM2^T^ formed a novel lineage, branching deeply before the divergence of the genera *Limibaculum, Thermohalobaculum* and *Rubrimonas* (Fig. S1, available in the online version of this article). NKW57^T^ exhibited close relationships with various species of the genus *Microbulbifer*, with sequence identities ranging from 92.0 to 94.9%. These values fell below the threshold for species delineation [34]. The phylogenetic tree based on the 16S rRNA gene sequence indicated that strain NKW57^T^ formed a novel lineage separate from the genus *Microbulbifer* (Fig. S2).

## Genomic properties

Genome sequencing was performed on the DNBSEQ-400 platform by BGI JAPAN (Kobe, Japan). Genome assembly was performed using Unicycler 0.5.0 in normal mode [35]. Genome annotation was performed using DFAST [36] and the analysis was conducted using KOFAMkoala version 2023-04-01 [37] and KEGG mapper [38]. Average nucleotide identity (ANI) was determined using the JSpeciesWS server [39]. Digital DNA–DNA hybridisation (dDDH) values were calculated using the Genome-to-Genome Distance Calculator (GGDC) version 3.0 [40,41]. Average amino acid identity (AAI) was calculated using the Kostas Lab AAI calculator [42]. The phylogenetic tree was reconstructed using the Up-to-date Bacterial Core Genome (UBCG) pipeline with the RAxML tool based on the amino acid sequences of 81 concatenated core genes [43] and visualised using the mega 11 software package [32,33]. Evolutionary distances (EDs) between NKW23^T^ and closely related strains were calculated with IQ-TREE based on the Le and Gascuel with empirical base frequencies a proportion of invariable sites and ten rate categories (LG+F+I+R10) model, with MSA files of bac120 marker genes generated using the easyCGTree pipeline [44,45]. The polysaccharide degradation activities of species of the genus *Microbulbifer* have been reported previously [14,15, 21, 46, 47] therefore, carbohydrate-active enzymes (CAZymes) in the genomes were examined using dbCAN meta server 2.0 [48]. Secondary metabolism biosynthetic gene clusters (BGCs) were screened using antiSMASH version 6.0, with default parameters [49].

The assembled genome of NKW23^T^ comprised 201 contigs, with a total sequence length of 5 505 440 bp, an N_50_ of 105 891 bp and 4891 protein-coding sequences (CDSs). The DNA G+C content was 71.4% and the depth coverage was 289× (Table S1). Within the genome of NKW23^T^, complete gene sets for eight central carbohydrate metabolism pathways, including glycolysis, gluconeogenesis, pyruvate oxidation, the TCA cycle and the pentose phosphate pathway, were discovered. The genome encoded a complete set of assimilatory nitrate reduction genes for nitrogen metabolism. In addition, a set of *SoxXYZABCD* genes involved in the SOX pathway, an oxidation pathway for inorganic sulphur compounds, was identified. The SOX pathway was also detected in phylogenetic relatives *L. halophilum* CAU 1123^T^, *L. sediminis* FT325^T^ and *T. xanthum* M0105^T^ (Table S2). Genes for oxidative phosphorylation, including succinate dehydrogenase (complex II), cytochrome reductase (complex III), cytochrome *c* oxidase (complex IV) and *cbb_3_*-type cytochrome *c* oxidase, were also encoded. The predicted BGCs of NKW23^T^ contained six gene clusters involved in the biosynthesis of terpenes, thioamides, aryl polyene, type one polyketides (T1PKS), type three polyketides (T3PKS) and *N*-acetyl glutaminyl glutamine amide (NAGGN) (Table S3). The predicted arylpolyene associated gene cluster showed a 10% amino acid similarity with oryzanaphthopyran A/oryzanaphthopyran B/oryzanaphthopyran C/oryzanthrone A/oryzanthrone B/chlororyzanthrone A/chlororyzanthrone B.

The assembled genome sequence of NKW57^T^ comprised 35 contigs, with a total sequence length of 3 593 200 bp, an N*_50_* of 728 696 bp and 3101 CDSs. The DNA G+C content was 58.8% and the depth coverage was 838× (Table S1). KEGG mapper analysis enabled the reconstruction of 59 complete KEGG modules, providing insights into the cellular functions of NKW57^T^. The genome was found to encode 11 complete gene sets for glycolysis, pyruvate oxidation, the TCA cycle, the pentose phosphate pathway, the Entner–Doudoroff pathway and the glyoxylate cycle. The predicted BGCs of NKW57^T^ contained four gene clusters involved in the biosynthesis of two ribosomally synthesised and post-translationally modified peptides (RiPPs), betalactone and non-ribosomal peptide synthase (NRPS)-like proteins (Table S4). One of the RiPPs-associated gene clusters showed 2% amino acid similarity with gausemycin A/gausemycin B, indicating that NKW57^T^ might function in bacterial interactions with brittle stars. Furthermore, the genome of NKW57^T^ included genes encoding carbohydrate-related enzymes, comprising five auxiliary activities (AAs), two carbohydrate-binding modules (CBMs), six carbohydrate esterases (CEs), fifteen glycoside hydrolases (GHs), eight glycosyltransferases (GTs) and two polysaccharide lyases (PLs). GH16, GH18 and GH19, which are involved in the degradation of polysaccharides such as agar, alginic acid, chitin and carrageenan, were not detected in the draft genome of NKW57^T^, but these GHs have been found in several strains of species of the genus *Microbulbifer* (Fig. S3). The genome of NKW57^T^ encoded fewer carbohydrate-related enzymes compared with those of members of the genus *Microbulbifer*, which utilises many polysaccharides as carbon sources and energy sources. This is consistent with the fact that NKW57^T^ did not degrade polysaccharides on agar media and utilised few of the available carbon sources.

**Fig. 1.**
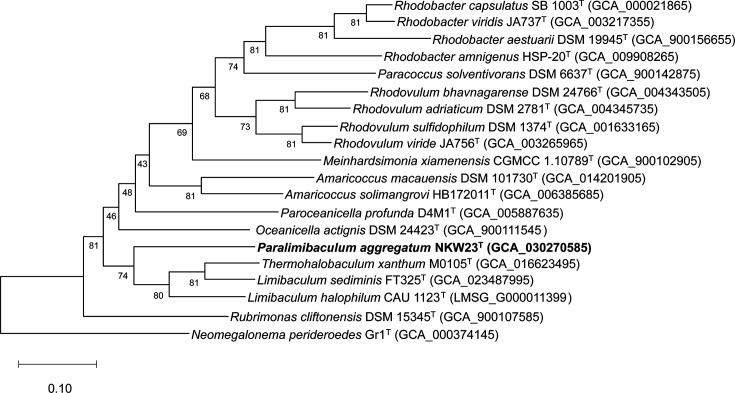
Maximum-likelihood phylogenetic tree based on the concatenated sequences of 81 core genes of representative members of the families *Paracoccaceae* and *Rhodobacteraceae*. GenBank accession numbers are given in parentheses. *Neomegalonema perideroedes* Gr1^T^ was used as the outgroup. Bar, 0.10 changes per nucleotide position. *This genomic data was obtained from the National Genomics Data Centre in PR China.

**Fig. 2.**
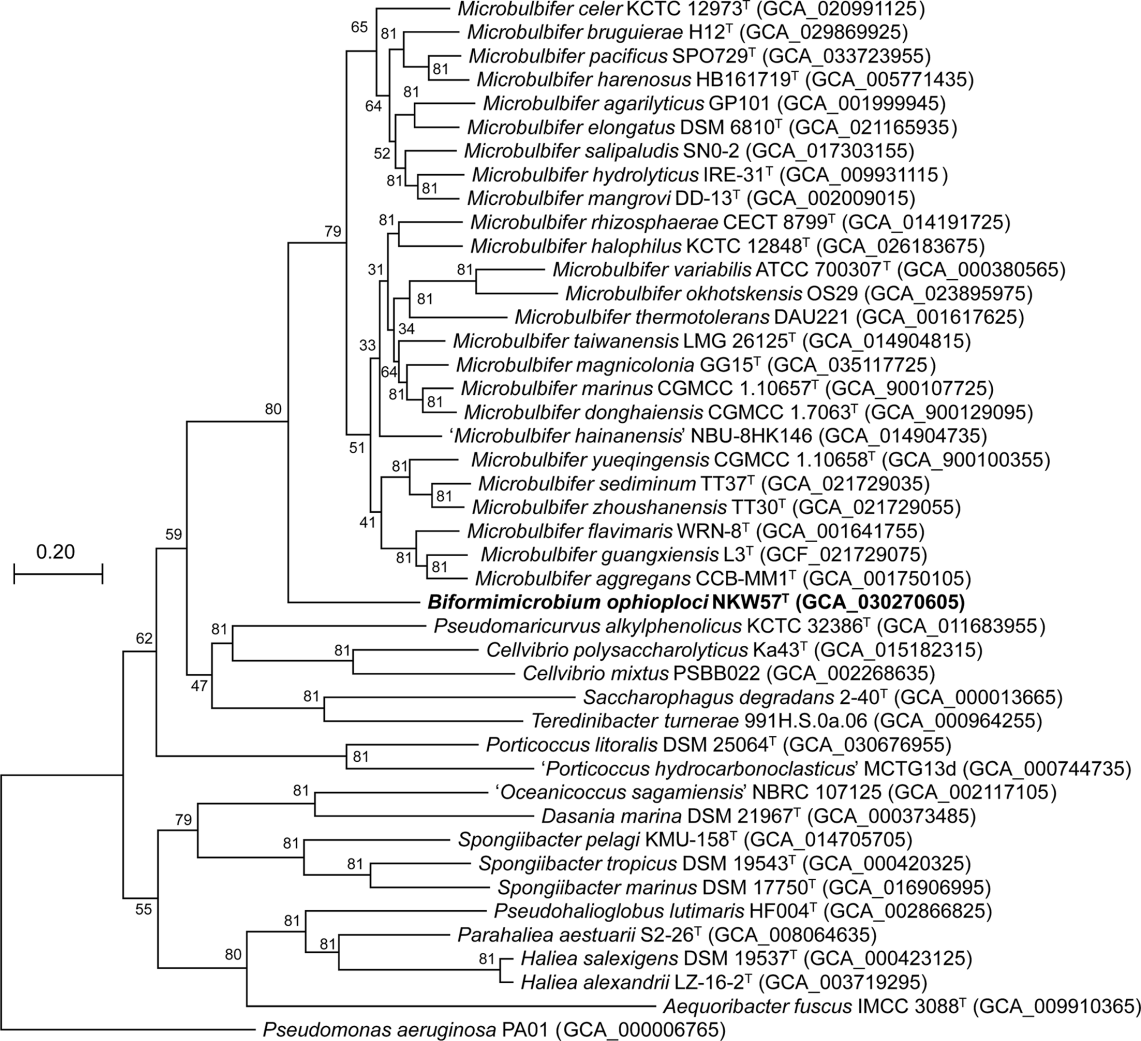
Maximum-likelihood phylogenetic tree based on concatenated sequences of 81 core genes of representative members of the genus *Microbulbifer* and other related genera. GenBank accession numbers are given in parentheses. *Pseudomonas aeruginos*a. PAO1 was used as an outgroup. Bar, 0.20 changes per nucleotide position.

The ANI and dDDH values between NKW23^T^ and its phylogenetic relatives from the family *Paracoccaceae* were 70.3–74.5% and 13.7–17.3%, respectively (Table S5). These values were below the commonly accepted thresholds for species delimitation [41,50]. AAI values between NKW23^T^ and members of the family *Paracoccaceae* ranged from 53.2 to 63.7% (Table S5), predominantly falling below the 60–80% range commonly used for genus delimitation, indicating a potential classification for NKW23^T^ as a member of a novel genus [51]. In the phylogenomic tree, NKW23^T^ branche into a group close to *L. halophilum* CAU 1123^T^, *L. sediminis* FT325^T^ and *T. xanthum* M0105^T^ (Fig. 1). The branching pattern was consistent with the phylogenetic relationships inferred from 16S rRNA gene sequences. Additionally, an ED threshold of 0.21–0.23 based on bac120 has been proposed as one standard for genus delimitation in the family *Paracoccaceae* [52]. The ED values of NKW23^T^ compared with *L. halophilum*, *L. sediminis* and *T. xanthum* were 0.32, 0.31 and 0.31, respectively (Table S6), exceeding the ED threshold values shown in previous studies. The ANI and dDDH values between NKW57^T^ and members of the genus *Microbulbifer* were 69.2–72.1% and 13.1–14.6%, respectively (Table S7). These values were substantially below the species delineation thresholds [41,50]. In addition, the AAI values between NKW57^T^ and species of the genus *Microbulbifer* were 62.7–65.9%, falling below the established genus delineation threshold (Table S7) [51]. For comparison, the intra-genus AAI values for members of the genus *Microbulbifer* varied between 67.3 and 89.4% (Table S8). The UBCG-derived phylogenomic tree indicated that NKW57^T^ is closely related to species of the genus *Microbulbifer* but forms a distinct branch separate from the three main clusters within the genus (Fig. 2). The AAI values of * M. variabilis* ATCC 700307^T^ and *M. okhotskensis* OS29^T^, when compared with other species of the genus *Microbulbifer*, were below 70% (Table S8). Despite this, these two strains were included in three clusters of members of the genus *Microbulbifer* (Fig. 2). Hierarchical clustering based on AAI values also segregated NKW57^T^ from the members of the genus *Microbulbifer* into a distinct clade (Fig. S4). The relationships depicted in the phylogenomic tree corresponded closely to those in the phylogenetic tree based on the 16S rRNA gene, further substantiated by the similarity in the ANI and AAI values (Fig. S2 and Table S7). Pangenome analysis of NKW57^T^ and species of the genus *Microbulbifer* was performed using the anvi’o package version 7 [53] and DIAMOND [54]. The anvi’ o pan-genomics pipeline successfully identified 17 438 gene clusters across 24 genomes. This included 1406 core gene clusters shared by all genomes and 10 172 singleton gene clusters (Fig. S5). The genome of NKW57^T^ contained 774 singleton gene clusters. In addition, 223 gene clusters, common to members of the genus *Microbulbifer* were absent in NKW57^T^.

## Physiology and chemotaxonomy

**Fig. 3.**
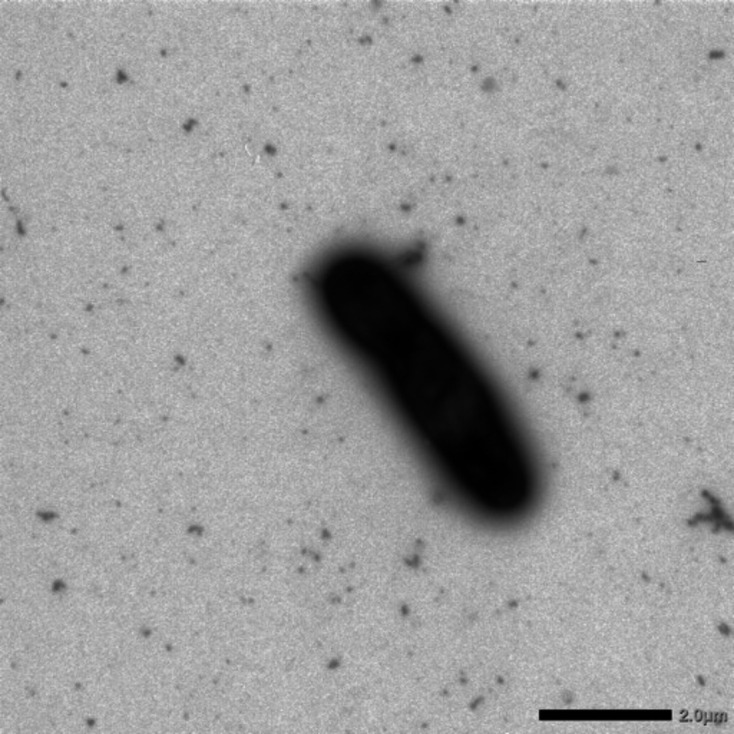
Transmission electron micrograph of NKW23^T^ after culture on MB agar medium at 37 °C for 72 h. Bar, 2.0 µm.

**Fig. 4.**
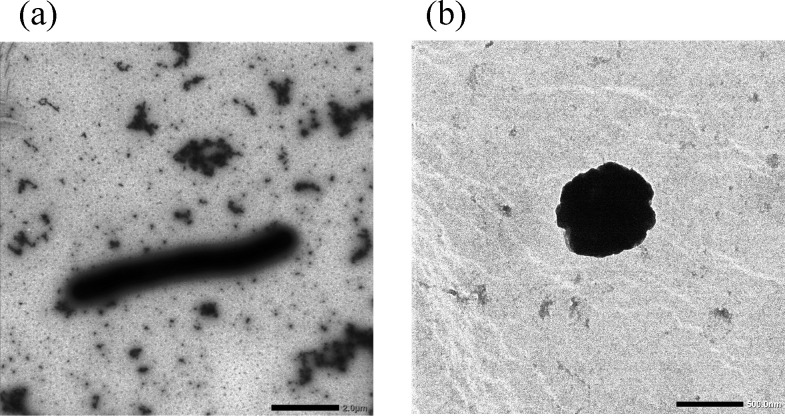
Transmission electron micrograph of NKW57^T^ (**a**) cultured on MB agar medium at 37 °C for 24 h. Bar, 2.0 µm, (**b**) cultured in MB agar medium at 37 °C for 96 h. Bbar,500 nm.

For transmission electron microscopy using a JEM-1400 (JEOL) microscope, cells of NKW23^T^ and NKW57^T^ were fixed in 2.0% glutaraldehyde for 8 h and then negatively stained with 2.0% uranyl acetate. Gram-staining was performed according to the Hucker method, and spore formation were checked as described previously [55]. Flagellar motility was assessed in semi-solid MB medium as described previously [16]. Cells of NKW23^T^ was characterised as Gram-stain-negative, non-spore-forming and non-motile rods, 0.7–1.2 µm in width and 3.0–4.4 µm in length (Fig. 3). The colonies were pale orange in colour. NKW23^T^ demonstrated cell aggregation in a liquid medium stationary phase (Fig. S6). These aggregates have not been reported in members of the genera *Limibaculum* and *Thermohalobaculum* [3,4, 10]. Cells of NKW57^T^ were also Gram-stain-negative and non-spore-forming, displaying no motility. Colonies of this strain were brown in colour. Cells in the exponential growth phase were rod-shaped, measuring 0.2–0.4 µm in width and 4.2–6.7 µm in length (Fig. 4). Upon reaching the stationary phase on both agar and liquid media, the cells transformed into cocci, ranging from 0.6 to 0.9 µm in diameter (Fig. 4).

The effects of temperature, NaCl and pH on the growth of NKW23^T^ and NKW57^T^ were assessed in MB liquid medium. Cell counts were conducted using cells stained with 5 ng µl^−1^ 4′,6-diamidino-2-phenylindole (DAPI) under an epifluorescence microscope, following methods from previous studies [56]. To determine the pH range for growth, the following buffer systems (Dojin Chemical Laboratory) were used at a final concentration of 100 mM at 37 °C: MES (pH 5.0–6.0), Bis–Tris (pH 6.5–7.0), HEPES (pH 7.5–8.0), Tricine (pH 8.5) and CHES (pH 9.0–10.0). The NaCl requirements were determined by varying the NaCl concentration in the medium from 0 to 12% (w/v) at 37 °C. Catalase and oxidase activities were examined as described previously [17]. The growth of NKW23^T^ occurred at pH 6.5–9.0 (optimum, pH 7.5), at 15–47 °C (optimum, 35–40 °C) and in the presence of 0–10% NaCl (optimum, 3–3.5%) (Fig. S7). In comparison with closely related species, no significant differences were observed in growth temperature and pH ranges; however, NKW23^T^ was capable of growing with up to 10% NaCl, whereas its phylogenetic relatives, i.e., *L. halophilum*, *L. sediminis* and *T xanthum*, could only grow with up to 7% NaCl (Table 1). Growth of NKW57^T^ occurred at pH 7.0–9.5 (optimum, pH 8.0) and at temperatures of 15–40 °C (optimum, 35–37 °C), in the presence of 1.0–5.0% NaCl (optimum, 2.5%) (Fig. S8). Regarding catalase and oxidase activities, NKW23^T^ tested positive for both, whereas NKW57^T^ was negative for catalase but positive for oxidase.

**Table 1.** Differential characteristics of NKW23^T^ and related strains Strains: 1, NKW23^T^ (this study); 2, *L. halophilum* CAU 1123^T^ [10]; 3, *L. sediminis* FT325^T^ [4]; 4, *T. xanthum* M0105^T^ [3]; 5, *C. acroporae* NCM2^T^ [6]. +, Positive; −, negative; w, weakly positive; nd, not determined.

Characteristic	1	2	3	4	5
Isolation origin	Brittle star	Reclaimed land mud	Mangrove sediment	Mangrove sediment	Scleractinian coral
Colony colour	Pale orange	Cream	Cream	Pale yellow	Pale yellow
Cell shape	Rods	Short rods	Ovoid, rods	Rods	Short rods
Flagellum	−	−	−	−	+
Motility	−	−	+	−	+
Catalase	+	+	+	+	+
Oxidase	+	+	+	−	+
Growth at/with:					
15 °C	+	−	−	+	−
45 °C	+	−	+	+	−
pH 6.0	−	−	+	+	−
pH 9.5	−	−	+	+	−
0% NaCl	+	+	+	−	−
7.5% NaCl	+	−	−	−	+
10% NaCl	+	−	−	−	+
Utilisation of					
d-mannitol	+	−	−	−	−
d-sorbitol	+	nd	nd	−	−
d-fructose	−	−	w	−	nd
d-mannose	+	−	−	−	+
Hydrolysis of					
gelatin	−	+	+	+	−
starch	−	−	+	−	nd
casein	−	−	−	nd	nd
Major fatty acids (>5%)	C_18 : 1_, C_16 : 0_, C_18 : 0_	C_18 : 1_ω7*c*, 11-methyl C_18 : 1_ ω7*c*	C_16 : 0_, C_18 : 1_ω7c, 11-methyl C_18 : 1_ω7*c*	C_16 : 0_ DMA, C_16 : 0_, C_18 : 1_ 7*c*,	C_16 : 0_, Summed feature 8*
Predominant polar lipids†	PE, PL, AL	PG, PE, APL. PC	PG, GL, PL, AL	DPG, PG, PC, PGL, PL, L, AL	PE, PG, AL, PL
DNA G+C content (%)‡	71.4	70.0	69.6	67.9	63.4

*Summed feature eight contains C_18 :  1_ω7*c* and/or C_18 :  1_ω6*c*.

†AL, aminolipid; APL, aminophospholipid; DPG, diphoshatidylglycerol; GL, glycolipid; L, lipid; PC, phosphatidylcholine; PE phosphatidylethanolamine; PG, phosphatidylglycerol; PGL, phosphoglycolipid; PL, phospholipid

‡DNA G+C contents of all strains were calculated from the genome data.

Carboxymethyl cellulose sodium salt (CMC), casein, alginic acid and starch degradation were tested using artificial seawater (ASW) agar plates containing 0.2% (w/v) of each substrate [57]. Urease activity was assessed using Stuart’s urea broth, supplemented with 3% (w/v) NaCl [58]. Hydrogen sulphide (H_2_S) production was confirmed as described previously [59]. The presence of DNase activity was checked on MB agar plates containing 0.2% (w/v) deoxyribonucleic acid from salmon sperm (Wako) and evaluated by applying 1 M HCl to the colonies. Gelatinase activity was evaluated using methods described previously [60]. The utilisation of carbon and energy sources was assessed using MB medium lacking peptone and yeast extract [61]. The medium was supplemented with a variety of carbon and energy sources, each added at a concentration of 2.0 g l^−1^. These carbon and energy sources included yeast extract, peptone, d-glucose, l-arabinose, d-xylose, d-mannose, maltose, d-fructose, lactose, sucrose, l-fucose, α-l-rhamnose, d-mannitol, d-sorbitol, glycerol, xylitol, inositol, trehalose, *N*-acetyl-d-glucosamine, methyl α-d-mannopyranoside, sodium citrate, fumaric acid, acetic acid, butyric acid, pyruvic acid, propionic acid, casamino acid and d/l-malic acid. Acid production from the carbon source was determined using marine oxidation fermentation (MOF) medium with a 0.2% (w/v) sugar source (d-glucose, l-arabinose, d-xylose, d-mannose, maltose, d-fructose, lactose, sucrose, l-fucose, α-l-rhamnose, d-mannitol, d-sorbitol, glycerol, xylitol, inositol, trehalose, *N*-acetyl-d-glucosamine and methyl α-d-mannopyranoside) [62,63]. Sulphur oxidation (Sox) activity of NKW23^T^ under aerobic conditions was measured using 96 h cultures grown in mixotrophic medium (MSTSY) supplemented with 2% NaCl at 37 °C [64]. Thiosulphate concentrations were measured in a high-pressure liquid chromatography system as described in previous studies [24]. *L. halophilum* CAU 1123^T^ was also examined for thiosulphate utilisation under the same conditions. NKW23^T^ grew on d-mannitol, d-sorbitol, glycerol, maltose, sucrose, d-mannose, fumaric acid, acetic acid, pyruvic acid, casamino acids and d/l-malic acid. NKW23^T^ utilised sugar alcohols, such as d-mannitol and d-sorbitol, but closely related species could not utilise these as carbon sources (Table 1). This strain produced acid only from mannitol. The DNase test result was negative, and no agar-degrading activity was observed. NKW57^T^ utilised yeast extract, peptone and casamino acid as carbon and energy sources. NKW57^T^ could not utilise d-glucose, d-mannose, *N*-acetyl-d-glucosamine and d/l-malic acid, which are utilised by the closely related species of the genus *Microbulbifer*, but used complex substrates, such as yeast extract, peptone and casamino acid, as energy sources (Table 2). Acid production was observed only for d-glucose and d-xylose (Table S9). For both strains, no degradation activity was observed for CMC, casein, starch, gelatin, agar, alginic acid and DNA (Tables1  2). NKW23^T^ was unable to utilise thiosulphate under aerobic conditions. No effect of thiosulphate on the growth of NKW23^T^ was observed.

**Table 2.** Differential characteristics of NKW57^T^ and strains of species of the genus *Microbulbifer* Strains*:* 1*,* NKW57^T^*;* 2, *Microbulbifer marinus* Y215^T^ [63]; 3*, Microbulbifer pacificus* SPO729^T^ [17,21]; 4*, Microbulbifer variabilis* Ni-2088^T^ [15]; 5, *Microbulbifer yueqingensis* Y226^T^ [63]; 6, *Microbulbifer harenosus* HB161719^T^ [21]; 7, *Microbulbifer rhizosphaerae* Cs16^T^ [18]; 8, *Microbulbifer echini* AM134^T^ [16]; 9, *Microbulbifer hydrolyticus* IRE-31^T^ [14,15, 17]. +, Positive; −, negative; w, weakly positive; nd, not determined.

Characteristic	1	2	3	4	5	6	7	8	9
Isolation origin	Brittle star	Marine sediment	Marine sponge	Marine algae	Marine sediment	Coastal sand	Rhizosphere of a halophyte	Sea urchin	Black liquor
Colony colour	Brown	Light yellow	Ivory	Milky white, yellowish green	Yellow	Cream	Sand yellow	Pale brown	Cream
Cell shape	Rods-cocci	Rods-cocci	Rods-cocci	Rods-cocci	Rods-cocci	Rods-cocci	Rods-cocci	Rods-cocci	Rods
Cell width (μm)	0.2–0.4	0.3–0.5	0.3–0.4	0.42–0.63	0.3–0.5	0.2–0.5	0.3–0.5	0.9–1.1	0.3–0.5
Cell length (μm)	4.2–6.7	2.5–5.0	4.0–6.0	3.5–13.5	2.5–5.0	2.0–7.0	2.0–4.0	3.3–5.5	1.1–1.7
Cocci cell (diameter, μm)	0.6–0.85	1.0–1.7×0.6–1.1	0.8–1.1	0.40–0.85	1.0–1.8×0.6–1.1	1.0–1.5×0.6–1.0	0.5–0.8	0.6–2.7	–
Growth at/with:									
10℃	–	–	–	–	–	–	–	+	+
45℃	–	–	+	–	+	+	–	–	–
pH 5.0	–	+	–	–	+	–	–	–	–
pH 10	–	+	+	–	+	+	+	–	–
0.5 % NaCl	–	–	–	+	+	–	–	–	–
10.0 % NaCl	–	–	–	–	+	+	–	–	–
Catalase	–	–	+	+	+	nd	+	+	+
Oxidase	+	+	+	+	+	+	+	+	+
Utilisation of									
glucose	–	+	+	+	+	+	+	nd	+
mannose	–	–	–	+	+	–	+	+	–
*N*-acetyl-d-glucosamine	–	–	–	+	–	+	+	–	+
malic acid	–	–	–	–	–	w	+	nd	+
Hydrolysis of									
DNA	–	–	+	+	–	nd	–	+	–
casein	–	+	+	+	+	nd	+	+	+
gelatin	–	–	–	+	–	–	+	+	+
starch	–	+	+	+	–	+	–	+	+
agar	–	–	–	+	–	–	–	–	–
Susceptibility to									
ampicillin	+	–	–	nd	–	nd	nd	nd	+
tetracycline	w	–	–	nd	–	nd	nd	nd	–
kanamycin	w	+	+	nd	–	nd	nd	nd	–
Major fatty acids (>10 %)	C_15 : 0_, C_16 : 0_, C_17 : 0_ cyclo	iso-C_15 : 0_, iso-C_17 : 1_ω9*c*	iso-C_15 : 0_, iso-C_17 : 0_, iso-C_17 : 1_ω9*c*	C_16 : 0_,C_18 : 1_ω7*c*	iso-C_15 : 0_, iso-C_17 : 1_ω9*c*	iso-C_15:0_,summed feature 3, summed feature 8, summed feature 9*	iso-C_15 : 0_,iso-C_17 : 1_ω8*c*,iso-C_11 : 0_ 3-OH, iso-C_17 : 0_	C_14 : 0_, C_16 : 0_, summed feature 8*	iso-C_15 : 0_, iso-C_17 : 1_ω9*c*
Predominant polar lipids	PE, PG, GL, APL	PE, PG, GL	PE, PG, PL, APL	PE, PG, PS	PE, PG, GL	PE, PG, GL, PL	PE, PG, PS, AGPL	PE, PS, APL, PL, AL	PE, PG
DNA G+C content (%)†	58.8	59.8	58.4	48.4	62.0	58.2	59.8	56.1‡	57.6

*Summed feature 3 contains C_16  :  1_ω7*c* and/or C_16  :  1_ω6*c*, summed feature eight contains C_18  :  1_ω6*c* and/or C_18  :  1_ω7*c*, summed feature 9 contains iso-C_17  :  1_ω9*c* and/or C_16  :  0_ 10-methyl

†These values were calculated from genome data, except for column 8

‡Data from Lee *et al.* [16]

Growth under anaerobic conditions was assessed by incubating the strains in MB liquid medium containing sodium nitrite (10 mM), potassium nitrate (10 mM), sodium thiosulphate (10 mM) and sodium sulphate (10 mM) as electron acceptors at 37 °C for 14 days. The gas phase was filled with 100% N_2_ and the culture tubes were tightly sealed with butyl rubber stoppers. Anaerobic growth was not observed in either of the strains. The reference strains *L. halophilum* CAU 1123^T^ and *M. marinus* CGMCC 1.10657^T^ were also examined for growth under anaerobic conditions, but no growth was observed. Susceptibility to antibiotics was tested in MB liquid medium, supplemented with the following antibiotics at the indicated final concentrations (μg ml^−1^): ampicillin (10), rifampicin (5), chloramphenicol (30), tetracycline (10), kanamycin (30) and streptomycin (10). The cultures were incubated at 37 °C for 3 days. NKW23^T^ was susceptible to kanamycin, streptomycin, tetracycline, chloramphenicol and rifampicin but exhibited resistance to ampicillin. NKW57^T^ was susceptible to ampicillin and chloramphenicol and showed weak sensitivity to kanamycin, streptomycin, tetracycline and rifampicin.

The intracellular fatty acid compositions of NKW23^T^ and NKW57^T^ were analysed using cells harvested in the late exponential growth phase. The extraction and analysis procedures followed those described in a previous study [56]. For NKW23^T^, the major fatty acids, constituting over 5% of the total amount, were C_18 : 1_ (55.7%), C_16 : 0_ (37.0%) and C_18 : 0_ (5.6%), which were similar to those found in closely related species (Table 1). In addition, minor amounts of C_14 : 0_ (1.3%) and C_16 : 1_ (0.4%) were detected. For NKW57^T^, the major fatty acids (>10%) included C_15 : 0_ (24.9%), C_16 : 0_ (13.6%) and C_17 : 0_cyclo (16.9%) and other fatty acids (<10%) included C_10 : 0_ (7.6%), C_14 : 0_OH (6.8%), C_14 : 0_ (1.4%), C_15 : 1_ (2.0%), C_16 : 1_ (8.9%), C_17 : 0_ (5.8%), C_18 : 1_ (9.4%), C_18 : 1_ (9.4%) and C_19 : 1_ (1.1%).

Isoprenoid quinones and polar lipids of NKW23^T^ and NKW57^T^ were extracted from 2 g of cells harvested after cultivation in MB medium at 37 °C for 24 h. The isoprenoid quinone analysis followed the methods of a previous study [56]. Polar lipids were identified using molybdic acid (for total lipids) and additional specific reagents for detecting functional lipids following a two-dimensional TLC method [55]. For comparison, cells of *L. halophilum* CAU 1123^T^ and *M. marinus* CGMCC 1.10657^T^ were also analysed. The predominant isoprenoid quinone of NKW23^T^ was identified as ubiquinone-10 (Q-10), which was also the major quinone in the related strains *L. halophilum* CAU 1123^T^, *L. sediminis* FT325^T^ and *T. xanthum* M0105^T^. The polar lipids in NKW23^T^ consist of phosphatidylethanolamine (PE), five unidentified phospholipids (PLs) and one unidentified aminolipid (AL) (Fig. S9). Notably, phosphatidylglycerol (PG) typically present in members of the genus *Limibaculum* and *T. xanthum*, was absent in NKW23^T^ (Table 1). For NKW57^T^, ubiquinone-8 (Q-8) was the major isoprenoid quinone, which is consistent with species of the genus *Microbulbifer*. The polar lipids were PE, PG, one aminophospholipid (APL), six unidentified PLs and one unidentified AL (Fig. S9). Both PE and PG, commonly found in members of the genus *Microbulbifer*, were present in NKW57^T^, but glycolipids were not detected in this strain (Table 2).

On the basis of these data, we propose two novel genera, *Paralimibaculum* gen. nov., within the family *Paracoccaceae,* and *Biformimicrobium* gen. nov. within the family *Microbulbiferaceae.* Previous studies have identified a diverse range of microorganisms from echinoderms, such as starfish, sea cucumbers and sea urchins [16,55, 65], but only a few have been isolated from brittle stars. The successful isolation of members of two novel genera from brittle stars in this study highlights the unexplored diversity of prokaryotes hosted by these ecologically significant organisms. Considering the challenging conditions in tidal pools, where brittle stars are exposed to significant physicochemical fluctuations, their symbiotic bacteria probably play an important role in supporting host adaptation and survival in dynamically changing environments.

## Description of *Paralimibaculum* gen. nov

*Paralimibaculum* (Pa.ra.li.mi.ba’cu.lum. Gr. prep. *para*, like; N.L. neut. n. *Limibaculum*, a bacterial genus; N.L. neut. n. *Paralimibaculum*, a genus like *Limibaculum*).

Gram-stain-negative, non-motile, non-spore-forming, aerobic and rod-shaped. Catalase and oxidase activities are positive. The colonies are circular and pale orange in colour. Cell aggregation is observed during cultivation in a liquid medium. The major cellular fatty acids (>5%) are C_18 : 1_, C_16 : 0_ and C_18 : 0_. The major polar lipids are PE, five unidentified PLs and one unidentified AL. The predominant isoprenoid quinone is Q-10. The type species is *Paralimibaculum aggregatum*.

## Description of *Paralimibaculum aggregatum* sp. nov

*Paralimibaculum aggregatum* (ag.gre.ga’tum, L. part. adj. *aggregatum*, joined together, referring to the observation that the cells aggregated in a liquid medium).

Aerobic, Gram-stain-negative, non-spore-forming and non-motile. The cells are rod shaped. The colonies are circular, raised, slightly hard and pale orange in colour. Growth occurs at pH 6.5–9.0 (optimum, pH 7.5), at 15–47 °C (optimum, 35–40 °C) and in the presence of 0–10% NaCl (optimum, 3–3.5%). Catalase and oxidase activities are positive. d-mannitol, d-sorbitol, glycerol, maltose, sucrose, d-mannose, fumaric acid, acetic acid, pyruvic acid, casamino acid and d/l-malic acid are assimilated as carbon and energy sources, but not d-glucose, l-arabinose, d-xylose, d-fructose, lactose, l-fucose, α-l-rhamnose, xylitol, inositol, trehalose, *N*-acetyl-d-glucosamine, methyl α-d-mannopyranoside, sodium citrate, butyric acid and propionic acid. CMC, casein, starch, gelatin, agar and DNA are not hydrolysed. H_2_S is not produced. Antibiotic susceptibility testing is positive for kanamycin, streptomycin, tetracycline, chloramphenicol and rifampicin, but negative for ampicillin.

The type strain NKW23^T^ (= JCM 36220^T^ =KCTC 8062^T^) was isolated from the tissue of the brittle star, *Ophioplocus japonicus*, which was captured from a tidal pool in Wakayama, Japan. The DNA GC content of the type strain is 71.4 %, calculated from the draft genome sequence. The 16S rRNA gene and draft genome sequence of NKW23^T^ have been deposited in the GenBank/ENA/DDBJ databanks under the accession numbers OQ678878 and BSYI00000000, respectively.

## Description of *Biformimicrobium* gen. nov

*Biformimicrobium* (Bi.for.mi.mi.cro'bi.um. L. masc. adj. *biformis*, two-shaped; N.L. neut. n. *microbium*, a microbe; N.L. neut. n. *Biformimicrobium*, a two-shaped microbe).

These cells are aerobic, Gram-stain-negative, non-spore-forming and non-motile. Cell morphology changes from rod to cocci depending on the culture conditions. The colonies are circular and brown in colour. The major fatty acids (>10) are C_15 : 0_, C_16 : 0_ and C_17 : 0_-cyclo, and the predominant quinone is Q-8. PE, PG, APL and six unidentified PLs are the major polar lipids. The type species is *Biformimicrobium ophioploci*.

## Description of *Biformimicrobium ophioploci* sp. nov

*Biformimicrobium ophioploci* (o.phi.o.plo’ci. N.L. gen. n. *ophioploci*, of the brittlestar *Ophioplocus japonicus*).

These cells are Gram-stain-negative, aerobic, non-spore-forming and non-motile. The cells are rod shaped (4.2–6.7 µm long and 0.2–0.4 µm wide) during the exponential growth phase, but cocci shaped (0.6–0.85 µm diameter) cells appear during the stationary phase. Colonies are circular, convex and brown. The cells can grow at pH 7.0–9.5 (optimum, pH 8.0) and at temperatures of 15–40 °C (optimum, 35–37 °C), in the presence of 1.0–5.0% NaCl (optimum, 2.5%). Catalase activity is negative, and oxidase activity is positive. CMC, casein, starch, gelatin, agar, alginic acid and DNA cannot be degraded. H_2_S is not produced. Assimilates yeast extract, peptone and casamino acid as carbon sources, but not d-glucose, l-arabinose, d-xylose, d-mannose, maltose, d-fructose, lactose, sucrose, l-fucose, α-l-rhamnose, d-mannitol, d-sorbitol, glycerol, xylitol, inositol, trehalose, *N*-acetyl-d-glucosamine, methyl α-d-mannopyranoside, sodium citrate, fumaric acid, acetic acid, butyric acid, pyruvic acid, propionic acid and d/l-malic acid. Acid production from d-glucose and d-xylose is observed. Antibiotic susceptibility is positive for ampicillin and chloramphenicol, and weakly positive for kanamycin, streptomycin, tetracycline and rifampicin.

The type strain NKW57^T^ (=JCM 36221^T^=KCTC 8063^T^) was isolated from the tissue of a brittle star, *Ophioplocus japonicus*, which was captured in a tidal poor at Wakayama, Japan. The DNA G+C content of the type strain is 58.8%, as calculated from the draft genome sequence.The 16S rRNA gene sequence and draft genome sequence of NKW57^T^ have been deposited in the GenBank/ENA/DDBJ databanks under the accession numbers OQ678879 and BSYJ00000000, respectively.

## supplementary material

10.1099/ijsem.0.006530 Uncited Supplementary Material 1.

## Abbreviations

AAI: average amino acid identity
AL: aminolipid
ANI: average nucleotide identity
APL: aminophospholipid
CDSs: coding sequences
CMC: carboxymethyl cellulose
ED: evolutionary distance
GGDC: genome-to-genome distance calculator
ML: maximum-likelihood
MP: maximum-parsimony
NJ: neighbor-joining
PE: phosphatidylethanolamine
PG: phosphatidylglycerol
PL: phospholipid
Q-8: ubiquinone-8
Q-10: ubiquinone-10
TLC: thin-layer chromatography
UBCG: up-to-data bacterial core genome
