# Supplementary material for: Paralimibaculum aggregatum gen. nov. sp. nov. and Biformimicrobium ophioploci gen. nov. sp. nov., two novel heterotrophs from brittle star Ophioplocus japonicus
Source: Int J Syst Evol Microbiol. 2024 Sep 26;74(9):006530. doi: 10.1099/ijsem.0.006530 (PMC11426391; doi:10.1099/ijsem.0.006530)
Supplement: Uncited Supplementary Material 1. [file ijsem-74-06530-s001.pdf]

International Journal of Systematic and Evolutionary Microbiology
Supplementary materials

Paralimibaculum aggregatum **gen. nov. sp. nov. and** ***Biformimicrobium ophioploci*** **gen. nov. sp. nov., two novel heterotrophs from brittle star** ***Ophioplocus japonicus***

Keisuke Kawano[1], Tatsuya Awano[2], Arata Yoshinaga[2], Junji Sugiyama[2], Shigeki Sawayama[1], Satoshi Nakagawa[1,3,4]

[1]Laboratory of Marine Environmental Microbiology, Division of Applied Biosciences, Graduate School of Agriculture, Kyoto University, Oiwake-cho, Kitashirakawa, Sakyo-ku, Kyoto 606-8502, Japan

[2]Laboratory of Tree Cell Biology, Division of Forest and Biomaterials Science, Graduate School of Agriculture, Kyoto University, Oiwake-cho, Kitashirakawa, Sakyo-ku, Kyoto 606-8502, Japan

[3]Institute for Extra-Cutting-Edge Science and Technology Avant-Garde Research (X-Star), Japan Agency for Marine-Earth Science & Technology (JAMSTEC), 2-15 Natsushima-Cho, Yokosuka 237-0061, Japan

[4]Section for Exploration of Life in Extreme Environments, Exploratory Research Center on Life and Living Systems (ExCELLS), National Institute of Natural Sciences, 5-1 Higashiyama, Myodaiji-Cho, Okazaki 444-8787, Japan

[*]Correspondence: Satoshi Nakagawa, nakagawa.satoshi.7u@kyoto-u.ac.jp

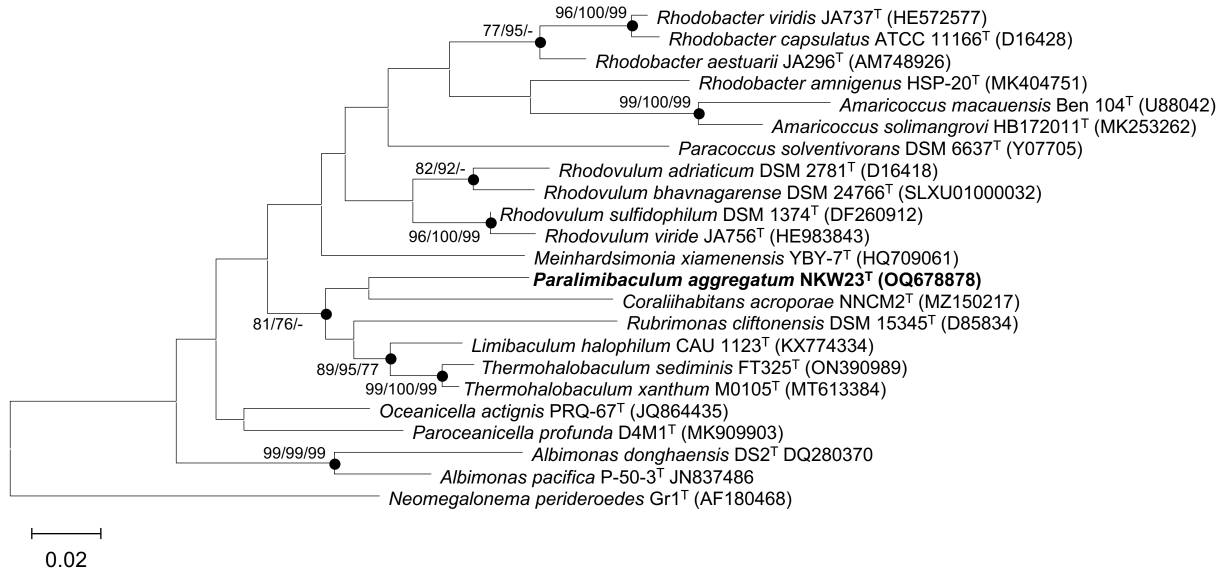

Fig. S1. Maximum-likelihood phylogenetic tree of strain NKW23^T based on the 16S rRNA gene sequences. Nodes with filled circles indicate branches that were also recovered with the neighbor-joining and maximum-parsimony methods with more than 70% bootstrap values. Bootstrap values are indicated at the reproducible nodes in the order of maximum-likelihood/neighbor-joining/maximum-parsimony methods. *Neomegalonema perideroedes* Gr1^T was used as an outgroup. Bar, 0.02 substitutions per nucleotide position.

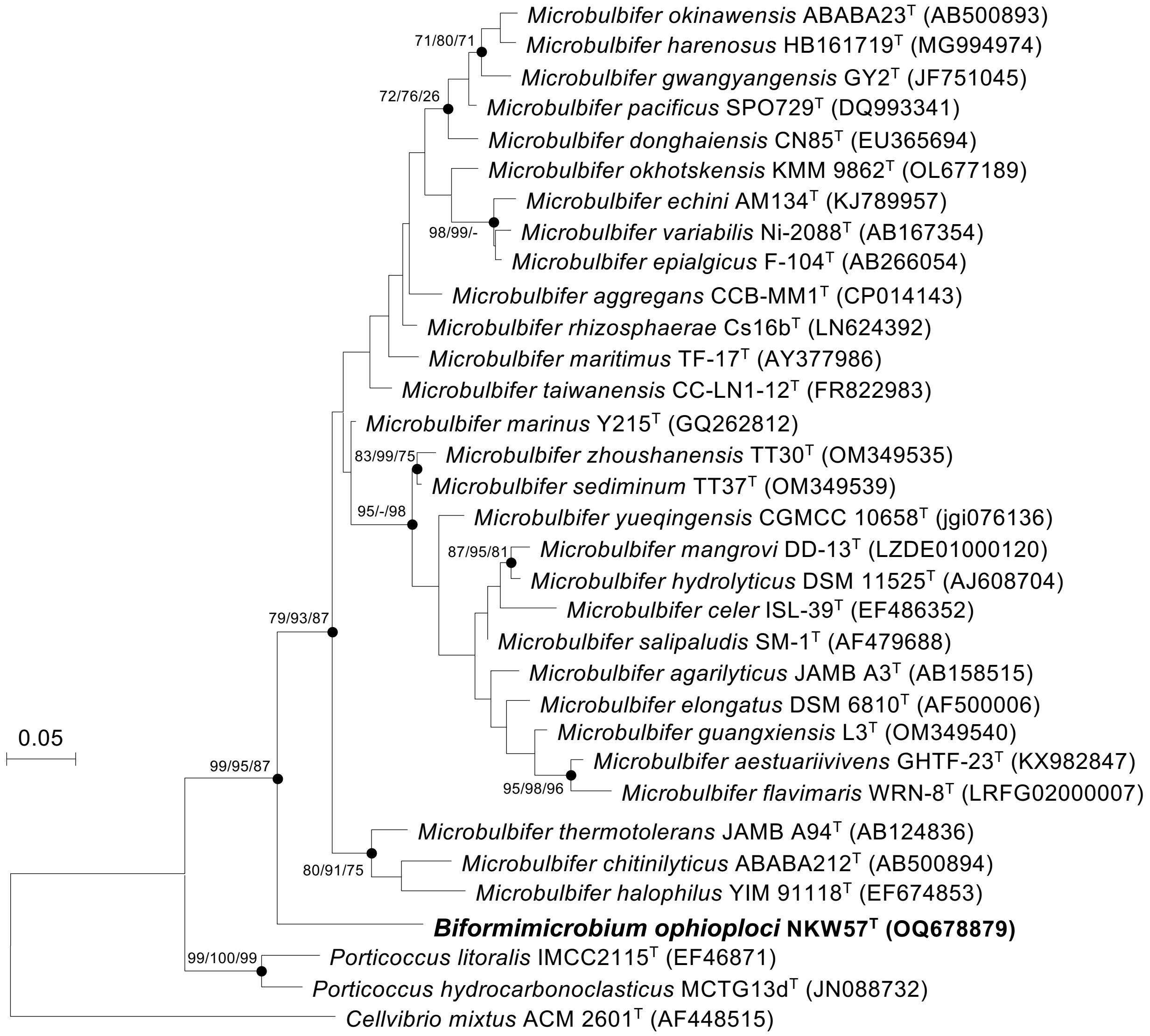

Fig. S2. Maximum-likelihood phylogenetic tree of strain NKW57^T based on the 16S rRNA gene sequences. Nodes with filled circles indicate branches that were also recovered with the neighbor-joining and maximum-parsimony methods with more than 70 % bootstrap values. Bootstrap values are indicated at the reproducible nodes in the order of maximum-likelihood/neighbor-joining/maximum-parsimony method. *Cellvibrio mixtus* ACM 2601^T was used as an outgroup. Bar, 0.05 substitutions per nucleotide position.

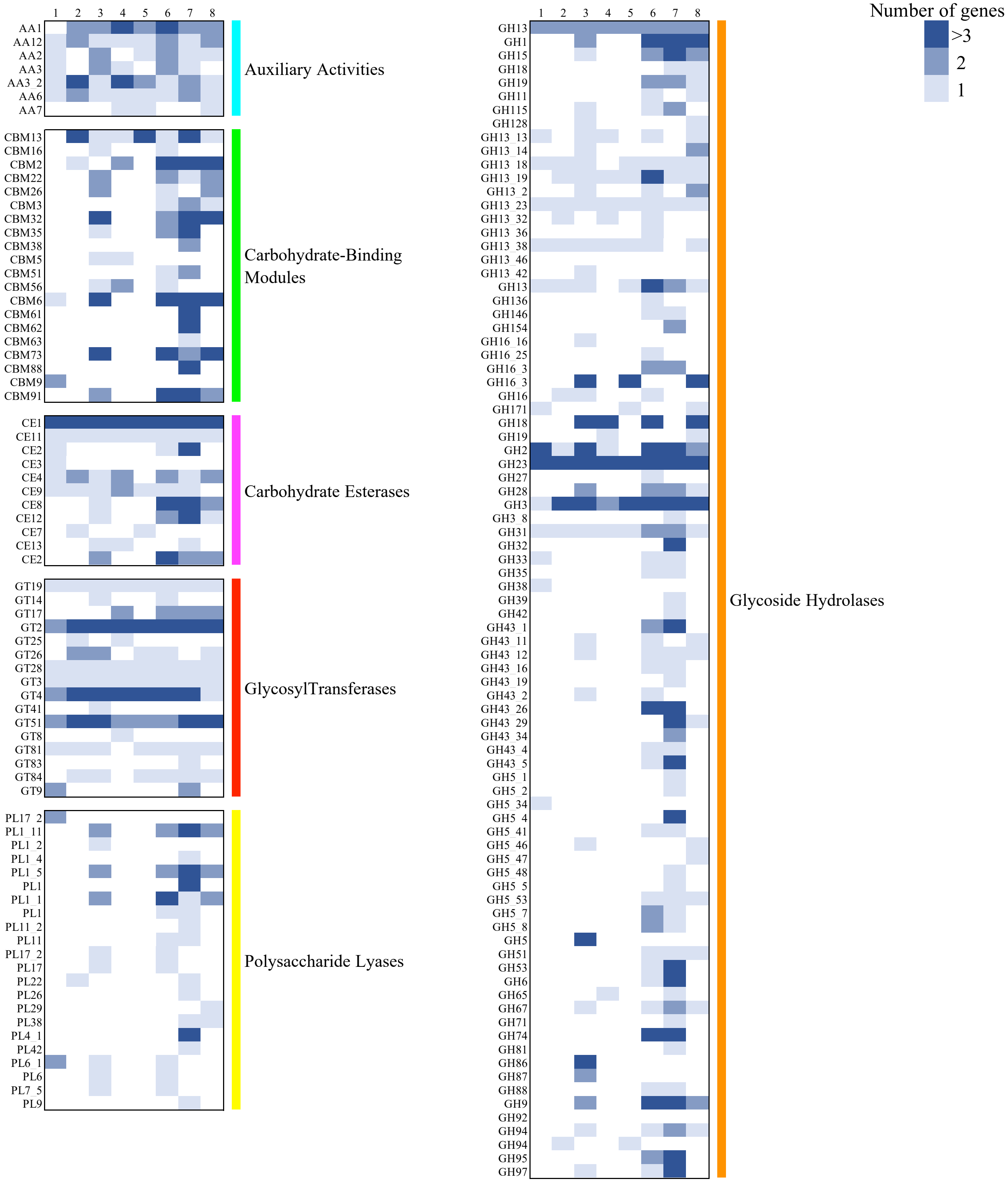

Fig. S3. CAZy carbohydrate active enzyme families found in genomes of the strain NKW57^T and compared with their closest phylogenetic relatives. Families have been divided by enzyme classes according to the CAZy database. Strains: 1, NKW57^T; 2, *Microbulbifer marinus* Y215^T; 3, *Microbulbifer pacificus* SPO729^T; 4, *Microbulbifer variabilis* Ni-2088^T; 5, *Microbulbifer yueqingensis* Y226^T; 6, *Microbulbifer harenosus* HB161719^T; 7, *Microbulbifer rhizosphaerae* Cs16^T; 8, *Microbulbifer hydrolyticus* IRE-31^T.

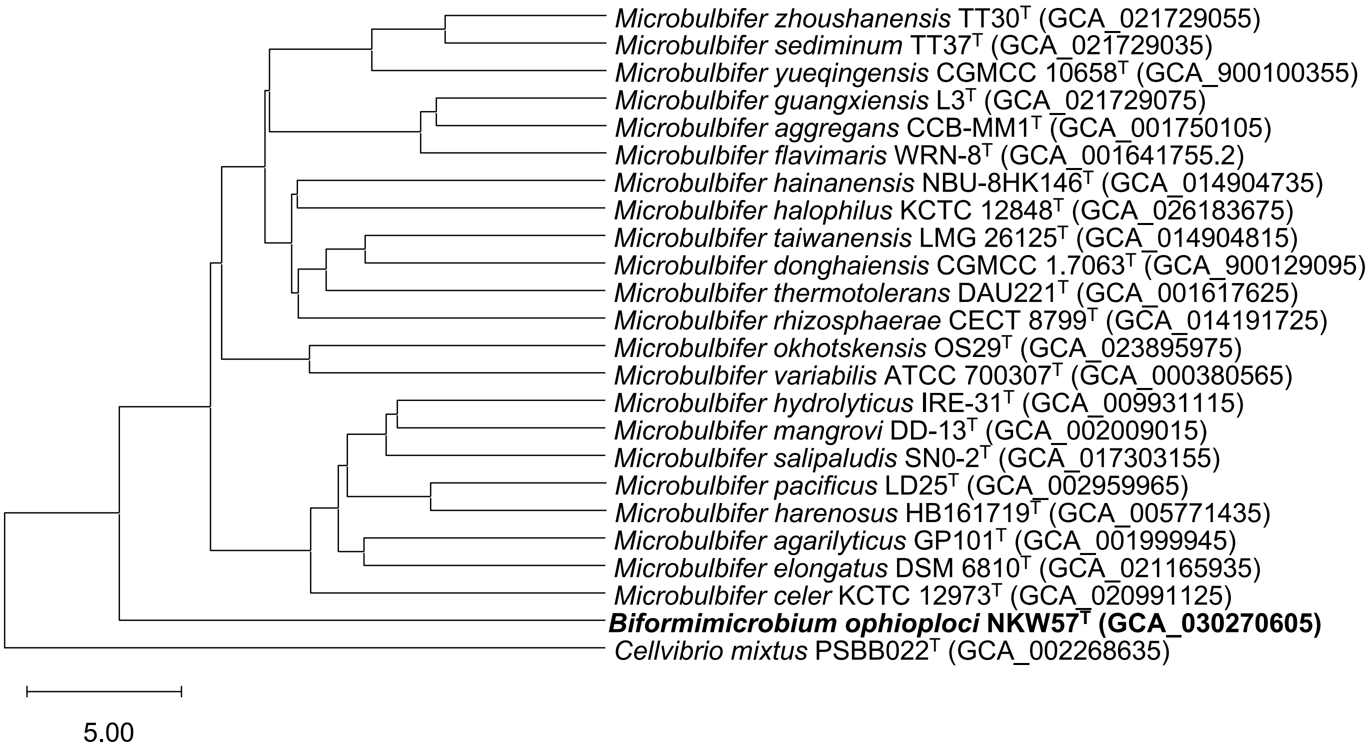

Fig. S4. Hierarchical clustering of taxa based on AAI values between strain NKW57^{T} and strains of the genus *Microbulbifer*

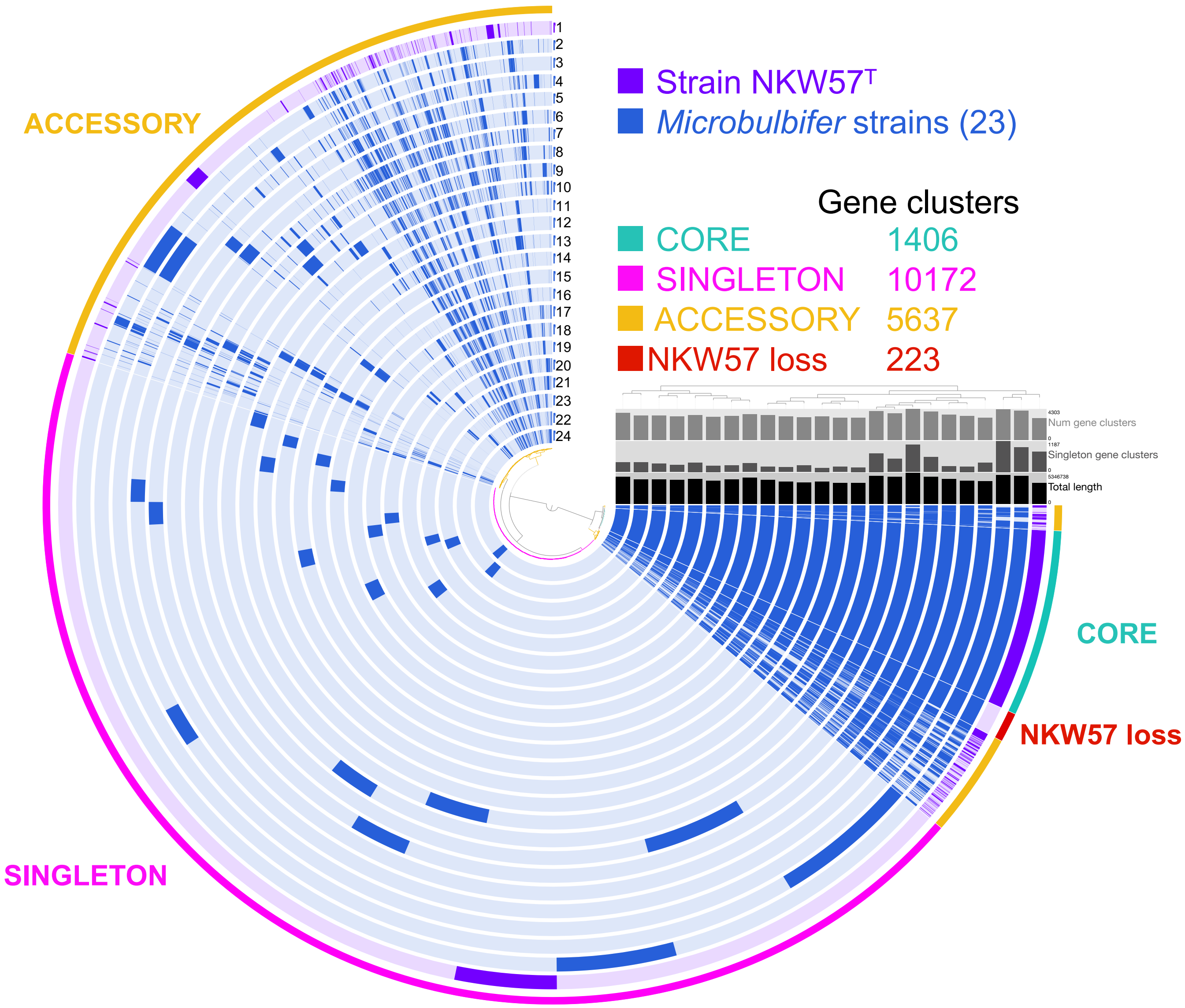

Fig. S5. The pan-genomic analysis of strain NKW23^{T} and the genus *Microbulbifer* strains. Bars represent the occurrence of gene clusters in a given genome. CORE (turquoise) indicates gene clusters, including SCG clusters common to all 24 strains. SINGLETON (pink) is the singleton gene cluster of each strain. ACCESSORY (orange) are gene clusters specific to some strains within the pan-genome. NKW57 loss (Red) is a gene cluster not conserved only in the strain NKW57^{T}. Strains: 1, NKW57^{T}; 2, *Microbulbifer variabilis* ATCC 700307^{T}; 3, *Microbulbifer okhotskensis* OS29^{T}; 4, *Microbulbifer thermotolerans* DAU221^{T}; 5, *Microbulbifer marinus* CGMCC 10657^{T}; 6, *Microbulbifer donghaiensis* CGMCC 1.7063^{T}; 7, *Microbulbifer hainanensis* NBU-8HK146^{T}; 8, *Microbulbifer rhizosphaerae* CECT 8799^{T}; 9, *Microbulbifer halophilus* KCTC 12848^{T}; 10, *Microbulbifer taiwanensis* LMG 26125^{T}; 11, *Microbulbifer flavimaris* WRN-8^{T}; 12, *Microbulbifer guangxiensis* L3^{T}; 13, *Microbulbifer aggregans* CCB-MM1^{T}; 14, *Microbulbifer yueqingensis* CGMCC 10658^{T}; 15, *Microbulbifer sediminum* TT37^{T}; 16, *Microbulbifer zhoushanensis* TT30^{T}; 17, *Microbulbifer mangrovi* DD-13^{T}; 18, *Microbulbifer hydrolyticus* IRE-31^{T}; 19, *Microbulbifer salipaludis* SN0-2^{T}; 20, *Microbulbifer celer* KCTC 12973^{T}; 21, *Microbulbifer elongatus* DSM 6810^{T}; 22, *Microbulbifer agarilyticus* GP101^{T}; 23, *Microbulbifer pacificus* LD25^{T}; 24, *Microbulbifer harenosus* HB161719^{T}.

(a) (b) (c)

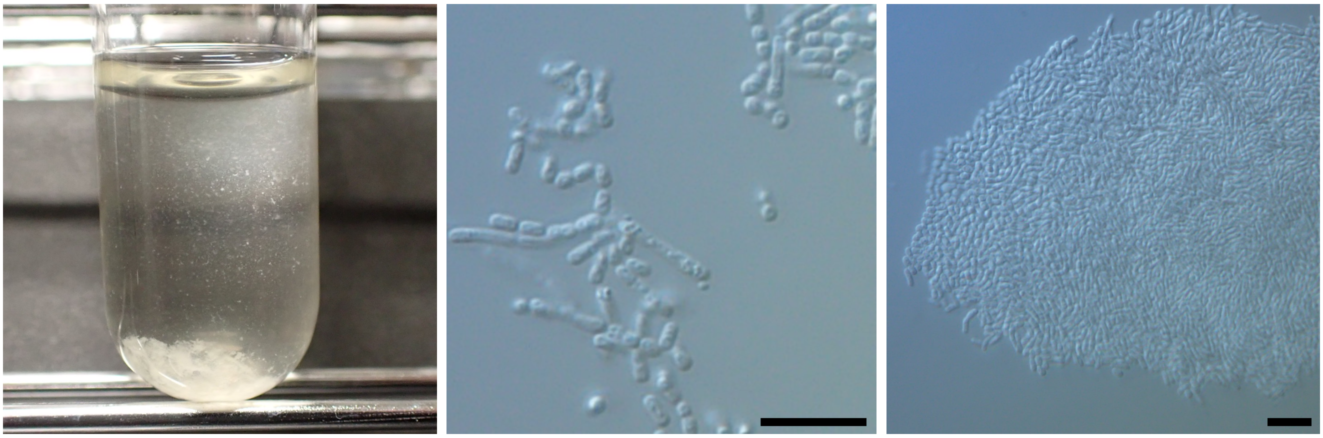

Fig. S6. (a) Cell aggregation of NKW23^{T} incubated in MB liquid medium at 37 °C for 72 hours. (b and c) Cell morphology of NKW23^{T} cultured in MB liquid medium under light microscopy. Bar, 10 μm.

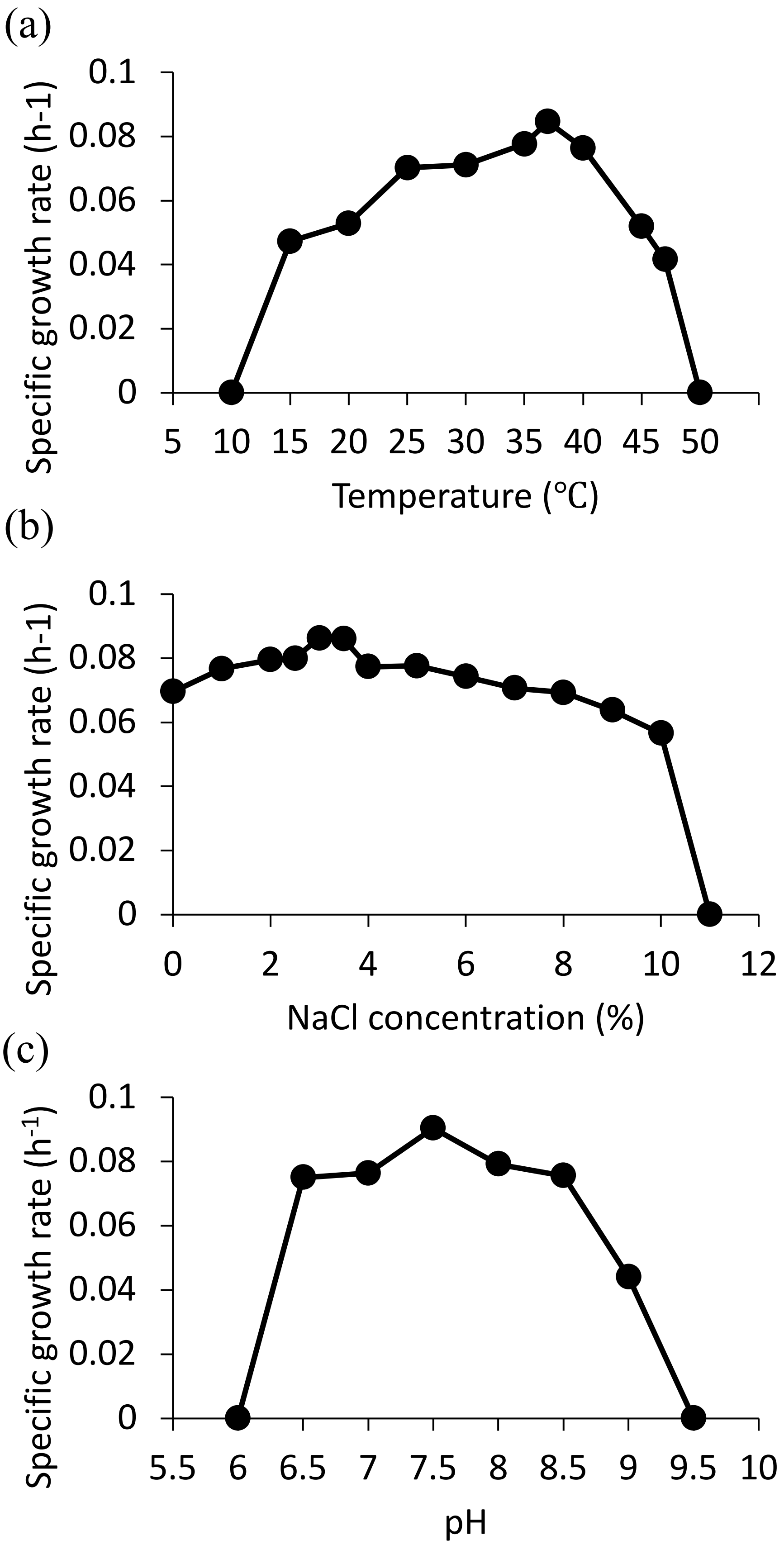

Fig. S7. Effects of temperature (a), NaCl concentration (b), and pH (c) on the growth of strain NKW23^{T}. Growth curves at different temperatures were determined in MB medium. The effect of NaCl concentration and pH was determined in MB medium at 37 °C.

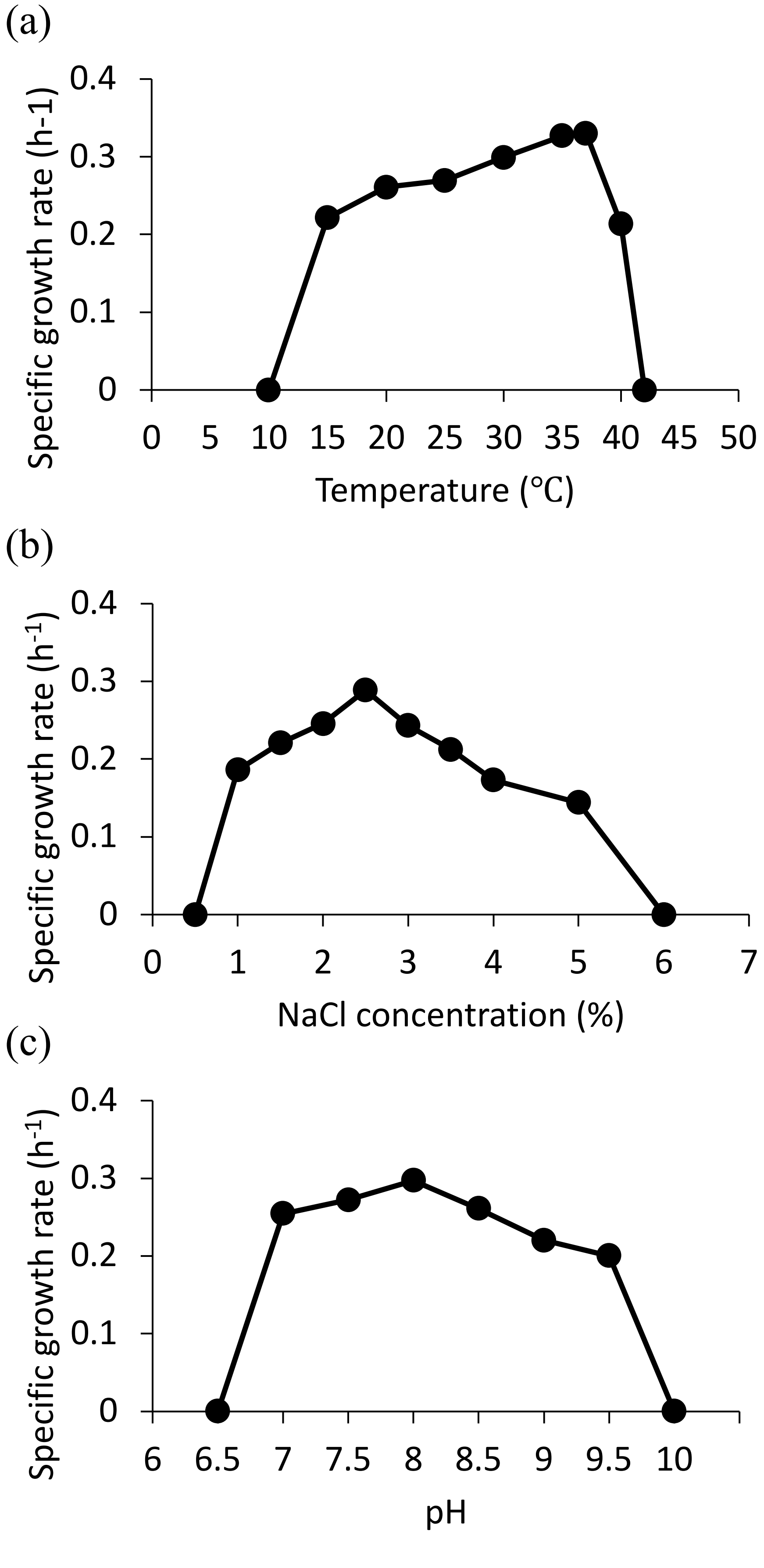

Fig. S8. Effects of temperature (a), NaCl concentration (b), and pH (c) on the growth of strain NKW57^{T}. Growth curves at different temperatures were determined in MB medium. The effect of NaCl concentration and pH was determined in MB medium at 37 °C.

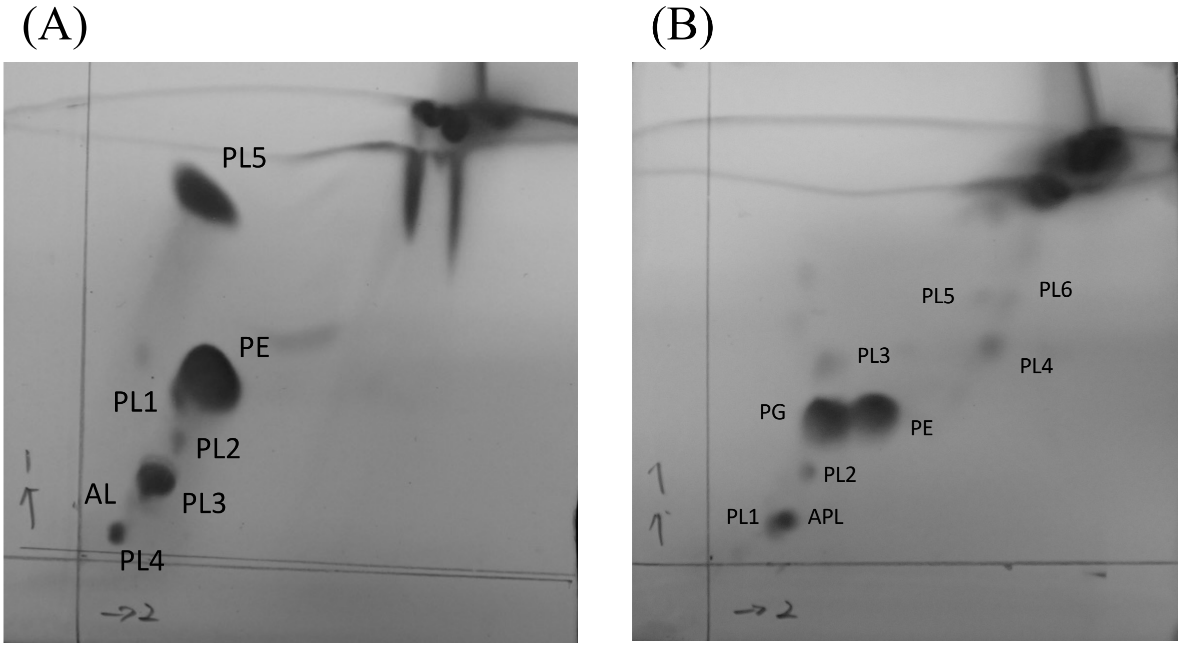

Fig. S9. Two-dimensional thin-layer chromatogram of polar lipids of (A) NKW23^T, (B) NKW57^T. PE, phosphatidylethanolamine; PG, phosphatidylglycerol; AL, unknown aminophospholipid; APL, unknown aminophospholipid; PL, unknown polar lipids.

Table S1. General genomic profile of strains NKW23^T and NKW57^T

Strain	NKW23^T	NKW57^T
Total sequence length (bp):	5,505,440	3,593,200
Number of contigs:	201	35
Longest contigs (bp):	277,516	792,260
N50 (bp):	105,891	728,696
Gap Ratio (%):	0	0
G+C content (%):	71.4	58.8
Depth of coverage (×):	289.0	838
Number of CDSs:	4891	3,101
Average protein length:	320.9	348.2
Coding ratio (%):	85.5	90.1
Number of rRNAs:	3	1
Number of tRNAs:	57	55
Number of CRISPRs:	7	1

Table S2. KEGG modules detected in genomes of strain NKW23^T and neighbor strains.

KEGG modules	NKW23^T	CAU 1123^T	FT325^T	M0105^T
**Carbohydrate metabolism**				
Central carbohydrate metabolism				
M00002 Glycolysis, core module involving three-carbon compounds (6)	+	+	+	+
M00003 Gluconeogenesis, oxaloacetate => fructose-6P (8)	+	+	+	+
M00307 Pyruvate oxidation, pyruvate => acetyl-CoA (3)	+	+	+	+
M00009 Citrate cycle (TCA cycle, Krebs cycle) (16)	+	+	+	+
M00010 Citrate cycle, first carbon oxidation, oxaloacetate => 2-oxoglutarate (3)	+	+	+	+
M00011 Citrate cycle, second carbon oxidation, 2-oxoglutarate => oxaloacetate (13)	+	+	+	+
M00007 Pentose phosphate pathway, non-oxidative phase, fructose 6P => ribose 5P (4)	+	+	+	+
M00005 PRPP biosynthesis, ribose 5P => PRPP (1)	+	+	+	+
Other carbohydrate metabolism				
M00552 D-galactonate degradation, De Ley-Doudoroff pathway, D-galactonate => glycerate-3P (5)	-	-	+	-
M00854 Glycogen biosynthesis, glucose-1P => glycogen/starch (4)	+	+	+	+
M00909 UDP-N-acetyl-D-glucosamine biosynthesis, prokaryotes, glucose => UDP-GlcNAc (5)	+	+	-	-
M00741 Propanoyl-CoA metabolism, propanoyl-CoA => succinyl-CoA (4)	+	+	+	+
**Energy metabolism**				
Carbon fixation				
M00168 CAM (Crassulacean acid metabolism), dark (2)	+	-	-	-
M00169 CAM (Crassulacean acid metabolism), light (2)	-	+	+	+
M00579 Phosphate acetyltransferase-acetate kinase pathway, acetyl-CoA => acetate (2)	-	-	+	+
Nitrogen metabolism				
M00531 Assimilatory nitrate reduction, nitrate => ammonia (3)	+	-	-	+
Sulfur metabolism				
M00595 Thiosulfate oxidation by SOX complex, thiosulfate => sulfate (7)	+	+	+	+
Photosynthesis				
M00597 Anoxygenic photosystem II (2)	-	+	+	-
ATP synthesis				
M00144 NADH:quinone oxidoreductase, prokaryotes (14)	-	+	-	-
M00149 Succinate dehydrogenase, prokaryotes (4)	+	+	+	+
M00151 Cytochrome bc1 complex respiratory unit (3)	+	+	+	+
M00155 Cytochrome c oxidase, prokaryotes (3)	+	+	+	+
M00156 Cytochrome c oxidase, cbb3-type (4)	+	+	+	+
M00157 F-type ATPase, prokaryotes and chloroplasts (8)	-	+	+	+
**Lipid metabolism**				
Fatty acid metabolism				
M00082 Fatty acid biosynthesis, initiation (6)	+	+	+	+
M00083 Fatty acid biosynthesis, elongation (6)	+	+	+	+
Lipid metabolism				
M00091 Phosphatidylcholine (PC) biosynthesis, PE => PC (1)	-	+	-	-
M00093 Phosphatidylethanolamine (PE) biosynthesis, PA => PS => PE (3)	-	+	-	-
**Nucleotide metabolism**				
Purine metabolism				
M00048 De novo purine biosynthesis, PRPP + glutamine => IMP (12)	+	+	+	+
M00049 Adenine ribonucleotide biosynthesis, IMP => ADP,ATP (4)	+	+	+	+
M00050 Guanine ribonucleotide biosynthesis, IMP => GDP,GTP (4)	+	+	+	+
M00958 Adenine ribonucleotide degradation, AMP => Urate (6)	-	-	+	+
M00959 Guanine ribonucleotide degradation, GMP => Urate (7)	-	-	+	+
Pyrimidine metabolism				
M00052 Pyrimidine ribonucleotide biosynthesis, UMP => UDP/UTP,CDP/CTP (3)	+	+	-	+
M00046 Pyrimidine degradation, uracil => beta-alanine, thymine => 3-aminoisobutanoate (4)	+	-	-	+
**Amino acid metabolism**				
Serine and threonine metabolism				
M00020 Serine biosynthesis, glycerate-3P => serine (3)	+	+	+	+
M00621 Glycine cleavage system (3)	+	+	+	+
M00033 Ectoine biosynthesis, aspartate => ectoine (5)	-	+	-	-
M00919 Ectoine degradation, ectoine => aspartate (4)	-	+	+	+
Cysteine and methionine metabolism				
M00021 Cysteine biosynthesis, serine => cysteine (2)	+	+	+	+
Branched-chain amino acid metabolism				
M00019 Valine/isoleucine biosynthesis, pyruvate => valine / 2-oxobutanoate => isoleucine (5)	+	+	+	+
M00570 Isoleucine biosynthesis, threonine => 2-oxobutanoate => isoleucine (6)	+	+	+	+
M00432 Leucine biosynthesis, 2-oxoisovalerate => 2-oxoisocaproate (4)	+	+	+	+
Lysine metabolism				
M00016 Lysine biosynthesis, succinyl-DAP pathway, aspartate => lysine (9)	+	+	+	+
M00957 Lysine degradation, bacteria, L-lysine => glutarate => succinate/acetyl-CoA (9)	-	-	-	+
Arginine and proline metabolism				
M00028 Ornithine biosynthesis, glutamate => ornithine (5)	+	+	+	+
M00844 Arginine biosynthesis, ornithine => arginine (3)	+	+	+	+
M00015 Proline biosynthesis, glutamate => proline (3)	+	+	+	+
M00970 Proline degradation, proline => glutamate (1)	+	+	+	+
Polyamine biosynthesis				
M00134 Polyamine biosynthesis, arginine => ornithine => putrescine (2)	-	-	+	+
Histidine metabolism				
M00026 Histidine biosynthesis, PRPP => histidine (10)	-	+	+	+
M00045 Histidine degradation, histidine => N-formiminoglutamate => glutamate (5)	+	-	-	-
Aromatic amino acid metabolism				
M00022 Shikimate pathway, phosphoenolpyruvate + erythrose-4P => chorismate (7)	+	+	+	+
M00023 Tryptophan biosynthesis, chorismate => tryptophan (7)	+	+	+	+
Other amino acid metabolism				
M00027 GABA (gamma-Aminobutyrate) shunt (5)	-	-	-	+
M00118 Glutathione biosynthesis, glutamate => glutathione (2)	+	-	+	+
**Metabolism of cofactors and vitamins**				
Cofactor and vitamin metabolism				
M00115 NAD biosynthesis, aspartate => quinolinate => NAD (5)	+	-	-	-
M00120 Coenzyme A biosynthesis, pantothenate => CoA (4)	+	+	+	+
M00881 Lipoic acid biosynthesis, plants and bacteria, octanoyl-ACP => dihydrolipoyl-E2/H (2)	+	+	+	+
M00880 Molybdenum cofactor biosynthesis, GTP => molybdenum cofactor (5)	+	+	+	+
M00140 C1-unit interconversion, prokaryotes (3)	+	+	+	+
M00924 Cobalamin biosynthesis, anaerobic, uroporphyrinogen III => sirohydrochlorin => cobyrinate a,c-diamide (13)	-	+	-	-
M00122 Cobalamin biosynthesis, cobyrinate a,c-diamide => cobalamin (9)	-	+	+	+
**Biosynthesis of terpenoids and polyketides**				
Polyketide sugar unit biosynthesis				
M00793 dTDP-L-rhamnose biosynthesis (4)	+	-	+	-
**Signature modules**				
**Module set**				
Metabolic capacity				
M00615 Nitrate assimilation (3)	+	-	-	+

* The numbers in parentheses indicate the number of genes constructing each module.
+, complete; -, not complete

Table S3. The predicted biosynthetic gene clusters (BGCs) of strain NKW23^T by using antiSMASH version 7.1.0

BCGs	Type	Length	Most similar known cluster	Similarity (%)
1	Arylpolyene, T3PKS	24,532	-	-
2	Thioamitides	19,202	-	-
3	T1PKS	43,891	-	-
4	NAGGN	14,735	-	-
5	Terpene	25,222	-	-
6	Arylpolyene	41,152	oryzanaphthopyran A/oryzanaphthopyran B /oryzanaphthopyran C/oryzanthrone A /oryzanthrone B/chlororyzanthrone A/chlororyzanthrone B	10

Table S4. The predicted biosynthetic gene clusters (BGCs) of strain NKW57^T by using antiSMASH version 7.1.0

BCGs	Type	Length	Most similar known cluster	Similarity (%)
1	Ripp-like	12,223	gausemycin A/gausemycin B	2
2	Betalactone	28,494	-	-
3	Ripp-like	10,896	-	-
4	NRPS-like, NRPS	59,365	capsular polysaccharide	11

Table S5. ANI, dDDH, and AAI values between strain NKW23^T and neighbor strains

Strain	Accession number of genome data	ANI (%)	dDDH (%)	AAI (%)
		with strain NKW23^T		
Limibaculum halophilum CAU 1123^T	LMSG_G000011399.1*	74.5	17.3	63.2
Thermohalobaculum sediminis FT325^T	JAMFKN000000000	73.8	16.9	63.0
Thermohalobaculum xanthum M0105^T	JAEHHL000000000	73.7	16.6	63.7
Coraliihabitans acroporae NCM2^T	JAHBMM000000000	71.8	14.4	61.5
Rubrimonas cliftonensis Och 317^T	FNQM00000000	71.9	14.8	57.4
Oceanicella actignis PRQ-67^T	FOHL00000000	71.8	14.9	59.5
Paroceanicella profunda D4M1^T	CP040818	71.5	14.5	56.2
Meinhardsimonia xiamenensis YBY-7^T	FNFV00000000	70.9	14.0	56.8
Paracoccus solventivorans ATCC 700252^T	FRCK00000000	70.3	13.7	53.2

*This genomic data was obtained from the National Genomics Data Center in China.

Table S6. Comparison of evolutionary distances (EDs) calculated by IQ-TREE 2

	1	2	3	4	5	6	7	8
**1. *Paralimibaculum aggregatum* NKW23^T**	-	-	-	-	-	-	-	-
2. *Limibaculum halophilum* CAU 1123^T	0.32	-	-	-	-	-	-	-
3. *Thermohalobaculum sediminis* FT325^T	0.31	0.17	-	-	-	-	-	-
4. *Thermohalobaculum xanthum* M0105^T	0.31	0.17	0.08	-	-	-	-	-
5. *Rubriomonas cliftonensis* DSM 15345^T	0.43	0.41	0.39	0.40	-	-	-	-
6. *Coraliihabitans acroporae* NCM2^T	0.34	0.31	0.31	0.31	0.45	-	-	-
7. *Oceanicella actignis* PRQ-67^T	0.42	0.38	0.38	0.38	0.40	0.42	-	-
8. Paroceanicella profunda D4M1^T	0.43	0.40	0.39	0.39	0.41	0.41	0.35	-

Table S7. ANI, dDDH, and AAI values between strain NKW57^T and strains of the genus *Microbulbifer*

Strain	Accession number of genome data	ANI (%)	dDDH (%)	AAI (%)
		with strain NKW57^T		
Microbulbifer marinus CGMCC 1.10657^T	GCA_900107725.1	72.1	14.3	65.9
Microbulbifer taiwanensis LMG 26125^T	GCA_014904815.1	72.1	14.2	64.0
Microbulbifer rhizosphaerae CECT 8799^T	GCA_014191725.1	72.1	13.7	64.2
Microbulbifer yueqingensis CGMCC 1.10658^T	GCA_900100355.1	72.1	14.5	65.5
Microbulbifer donghaiensis CGMCC 1.7063^T	GCA_900129095.1	72.0	14.3	65.7
Microbulbifer zhoushanensis TT30^T	GCA_021729055.1	71.9	14.6	64.8
Microbulbifer salipaludis SN0-2^T	GCA_017303155.1	71.9	14.5	65.5
Microbulbifer guangxiensis L3^T	GCA_021729075.1	71.8	14.3	65.4
Microbulbifer sediminum TT37^T	GCA_021729035.1	71.7	13.9	64.7
Microbulbifer hainanensis NBU-8HK146^T	GCA_014904735.1	71.6	14.3	64.3
Microbulbifer harenosus HB161719^T	GCA_005771435.1	71.5	13.9	63.9
Microbulbifer flavimaris WRN-8^T	GCA_001641755.2	71.5	14.3	65.6
Microbulbifer celer KCTC 12973^T	GCA_020991125.1	71.4	13.7	63.9
Microbulbifer hydrolyticus IRE-31^T	GCA_009931115.1	71.4	13.9	64.4
Microbulbifer aggregans CCB-MM1^T	GCA_001750105.1	71.4	14.1	65.8
Microbulbifer mangrovi DD-13^T	GCA_002009015.1	71.4	13.7	64.3
Microbulbifer pacificus LD25^T	GCA_002959965.1	71.3	13.9	63.9
Microbulbifer elongatus DSM 6810^T	GCA_021165935.1	71.3	13.8	64.1
Microbulbifer halophilus KCTC 12848^T	GCA_026183675.1	71.2	14.1	63.8
Microbulbifer agarilyticus GP101^T	GCA_001999945.1	71.0	13.8	65.2
Microbulbifer thermotolerans DAU221^T	GCA_001617625.1	70.1	13.4	65.1
Microbulbifer variabilis ATCC 700307^T	GCA_000380565.1	69.3	13.2	63.1
Microbulbifer okhotskensis OS29^T	GCA_023895975.1	69.2	13.1	62.7

Table S8. The result of AAI values between NKW57^T and strains of the genus *Micorobulbifer*

	1	2	3	4	5	6	7	8	9	10	11	12	13	14	15	16	17	18	19	20	21	22	23	24
**1. *Biformimicrobium ophioploci* NKW57^T**	100.0																							
2. *Microbulbifer marinus* CGMCC 1.10657^T	65.9	100.0																						
3. *Microbulbifer taiwanensis* LMG 26125^T	64.0	82.5	100.0																					
4. *Microbulbifer rhizosphaerae* CECT 8799^T	64.2	77.9	77.0	100.0																				
5. *Microbulbifer yueqingensis* CGMCC 1.10658^T	65.5	77.4	75.4	74.8	100.0																			
6. *Microbulbifer donghaiensis* CGMCC 1.7063^T	65.7	89.4	82.1	77.2	76.5	100.0																		
7. *Microbulbifer zhoushanensis* TT30^T	64.8	76.7	75.0	73.6	83.7	76.2	100.0																	
8. *Microbulbifer salipaludis* SN0-2^T	65.5	73.9	72.7	71.8	72.4	73.5	71.8	100.0																
9. *Microbulbifer guangxiensis* L3^T	65.4	77.1	75.9	74.2	76.7	76.6	76.4	72.8	100.0															
10. *Microbulbifer sediminum* TT37^T	64.7	76.9	75.5	73.8	84.0	76.2	89.2	72.0	76.6	100.0														
11. *Microbulbifer hainanensis* NBU-8HK146^T	64.3	79.5	77.3	75.9	75.6	78.8	75.5	72.3	75.7	75.5	100.0													
12. *Microbulbifer harenosus* HB161719^T	63.9	73.2	71.8	71.5	72.0	73.1	71.2	80.7	71.6	71.2	71.8	100.0												
13. *Microbulbifer flavimaris* WRN-8^T	65.6	77.6	76.4	74.9	77.1	77.1	76.7	72.9	87.1	76.8	75.9	71.9	100.0											
14. *Microbulbifer celer* ISL-39^T	63.9	73.7	72.8	71.6	72.4	73.0	71.6	78.8	71.9	71.7	72.4	79.1	72.3	100.0										
15. *Microbulbifer hydrolyticus* IRE-31^T	64.4	74.0	72.8	72.1	72.3	73.4	71.9	84.7	72.3	72.3	72.6	81.8	72.7	80.1	100.0									
16. *Microbulbifer aggregans* CCB-MM1^T	65.8	77.6	76.1	74.6	77.2	77.1	77.0	72.8	88.5	77.0	75.9	71.9	88.0	72.5	72.9	100.0								
17. *Microbulbifer mangrovi* DD-13^T	64.3	73.6	72.4	71.9	72.1	73.2	72.0	84.4	72.0	72.2	72.8	81.6	72.4	80.0	85.4	72.5	100.0							
18. *Microbulbifer pacificus* LD25^T	63.9	73.8	72.8	71.4	72.1	73.3	71.3	81.2	71.9	71.7	72.4	87.1	72.3	78.9	81.6	72.4	81.7	100.0						
19. *Microbulbifer elongatus* DSM 6810^T	64.1	73.3	72.3	71.3	71.9	72.9	71.1	82.4	72.0	71.3	71.5	80.5	72.3	78.9	81.9	72.2	82.2	81.3	100.0					
20. *Microbulbifer halophilus* KCTC 12848^T	63.8	78.3	77.4	76.4	75.7	77.5	75.4	72.0	75.4	75.9	77.2	71.9	76.0	73.1	72.4	75.4	72.4	71.7	72.0	100.0				
21. *Microbulbifer agarilyticus* GP101^T	65.2	72.3	70.7	70.1	71.0	71.9	70.5	80.9	71.3	70.6	70.8	78.7	71.6	77.0	80.2	71.9	80.2	79.2	82.8	70.5	100.0			
22. *Microbulbifer thermotolerans* DAU221^T	65.1	80.0	80.0	76.4	75.5	79.5	74.5	72.1	75.2	74.8	76.6	72.1	75.4	71.9	72.5	75.5	72.6	72.5	72.2	76.7	71.1	100.0		
23. *Microbulbifer variabilis* ATCC 700307^T	63.1	74.9	72.7	70.3	71.1	74.9	70.7	69.4	71.5	71.2	71.3	68.2	71.8	68.7	68.6	71.7	68.6	68.6	68.8	70.7	68.5	73.0	100.0	
24. *Microbulbifer okhotskensis* OS29^T	62.7	73.8	71.5	69.4	70.3	73.5	69.7	68.2	70.7	70.0	70.6	67.3	70.8	67.9	67.9	70.4	67.6	67.8	67.8	69.9	67.3	72.0	78.8	100.0

Table S9. Utilization and acid production from carbon source of strain NKW23^T and NKW57^T.

Carbon sources	Utilization		Acid production	
	NKW23^T	NKW57^T	NKW23^T	NKW57^T
D-glucose	-	-	-	+
L-arabinose	-	-	-	-
D-xylose	-	-	-	+
D-mannose	+	-	-	-
D-maltose	+	-	-	-
D-fructose	-	-	-	-
lactose	-	-	-	-
sucrose	+	-	-	-
L-fucose	-	-	-	-
α-L-rhamnose	-	-	-	-
D-mannitol	+	-	+	-
D-sorbitol	+	-	-	-
glycerol	+	-	-	-
xylitol	-	-	-	-
inositol	-	-	-	-
trehalose	-	-	-	-
N-acetyl-D-glucosamine	-	-	-	-
methyl α-D-mannopyranoside	-	-	-	-
sodiumcitrate	-	-	-	-
fumaric acid	+	-	-	-
acetic acid	+	-	-	-
butyric acid	-	-	-	-
pyruvic acid	+	-	-	-
propionic acid	-	-	-	-
casamino acid	+	+	-	-
D/L-malic acid	+	-	-	-
yeast extract	+	+	ND	ND
peptone	+	+	ND	ND

+, positive; −, negative; ND, not determined.
